# Down‐Regulation of TFEB With Defective Autophagy in the Susceptibility of Aging Kidneys to Septic Acute Kidney Injury

**DOI:** 10.1111/acel.70644

**Published:** 2026-07-21

**Authors:** Yu Xiang, Ying Fu, Zhiwen Liu, Yu Han, Wenwen Wu, Juan Cai, Dongshan Zhang, Zheng Dong

**Affiliations:** ^1^ Department of Nephrology, Hunan Key Laboratory of Kidney Disease and Blood Purification The Second Xiangya Hospital of Central South University Changsha China; ^2^ Department of Critical Medicin The Second Xiangya Hospital, Central South University Changsha Hunan China; ^3^ Department of Cellular Biology and Anatomy Medical College of Georgia at Augusta University and Charlie Norwood VA Medical Center Augusta Georgia USA

**Keywords:** acute kidney injury, aging, autophagy, sepsis, TFEB

## Abstract

Sepsis‐associated acute kidney injury (SA‐AKI) is a common and devastating disease that has a significantly higher incidence and greater severity in elderly patients, but the molecular basis underlying SA‐AKI in the elderly is largely unknown. Recent studies have proved autophagy as an intrinsic protective mechanism against AKI; however, the role and regulation of autophagy in aging kidneys remain unclear. Here we demonstrate that defective autophagy activation in aging kidneys is a key to their susceptibility to SA‐AKI. In our experiments, the ability of autophagy activation was impaired in aging kidneys in response to SA‐AKI in mice. In vitro, activation of autophagy with TAT‐Beclin‐1 peptide mitigated lipopolysaccharide (LPS)‐induced apoptosis and inflammation in senescent renal proximal tubular cells. Single‐cell sequencing revealed significant age‐related alterations in autophagy‐associated genes in septic AKI, including TFEB. Overexpression of TFEB could partially restore autophagic activity in senescent renal tubular cells and protect them from LPS‐induced damage. Moreover, in vivo treatment with the curcumin analog C1 (a TFEB activator) enhanced autophagic function in aging kidneys and reduced LPS‐induced AKI. These results demonstrate the defective autophagy activation in aging kidneys, which contributes to the SA‐AKI sensitivity and susceptibility in the elderly, suggesting a therapeutic strategy by enhancing autophagy.

## Graphical abstract

Aging is associated with impaired TFEB‐dependent autophagy in renal proximal tubular epithelial cells, leading to increased susceptibility to LPS‐induced injury and inflammation. Restoration of autophagy through TFEB activation or Tat–Beclin 1 treatment partially rescues autophagic function and confers protection against elderly sepsis‐associated acute kidney injury.

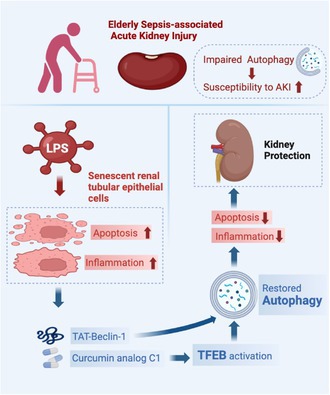

**Abbreviations**
ATG5: autophagy‐related 5
BECN1: beclin 1
BUMPT: The Boston University mouse proximal tubular cell line
BUN: blood urea nitrogen
C1: curcumin analog C1
Chloroquine: CQ
LPS: lipopolysaccharide
mPTECs: Murine primary tubular epithelial cells
RAB7: member RAS oncogene family 7
SA‐AKI: Sepsis‐associated acute kidney injury
SA‐β‐gal: Senescence‐associated galactosidase beta 1
SMAD3: SMAD family member 3
SQSTM1/p62: sequestosome 1
TFEB: Transcription factor EB

## 1 Introduction

Aging is an independent risk factor for acute kidney injury (AKI) (Perschinka et al. 2023). In histology, aging kidneys exhibit a reduction in nephron count, glomerular sclerosis, tubulointerstitial changes, and basement membrane thickening (Fang et al. 2020; O'Sullivan et al. 2017; Yamamoto and Isaka 2024). At the molecular level, aging is associated with various physiological, cellular, and epi/genetic alterations, as well as dysregulation of multiple intracellular signaling pathways (O'Sullivan et al. 2017). These changes collectively lead to increased susceptibility to injury and impaired kidney repair. Epidemiological studies indicate that the incidence of AKI rises progressively with age (Hsu et al. 2013). Moreover, elderly patients tend to experience more severe disease manifestations, with a higher likelihood of progression to chronic kidney disease (Hsu et al. 2013). Septic AKI is a prevalent form of AKI in intensive care units (Hato and Dagher 2025; Pais et al. 2024; Uchino et al. 2005). Its pathogenesis is highly complex, and the therapeutic window is often difficult to define (Hato and Dagher 2025). In elderly patients, it often results in more pronounced systemic inflammatory responses and exacerbated renal dysfunction, contributing to significantly higher mortality rates (Rex et al. 2023), but the underlying mechanism remains unclear. Moreover, despite its severity and rapid progression, there are currently no effective strategies for the prevention or treatment of this critical condition.

Autophagy is known to be an intrinsic protective mechanism against AKI (Li et al. 2025; Tang et al. 2020; Xiang et al. 2023). In this regard, we and others have demonstrated autophagy activation in septic AKI models where autophagy protects kidney tubules from septic injury (Leventhal et al. 2016; Mei et al. 2016). However, the role of autophagy in aging kidneys and their AKI susceptibility is largely unknown. There is evidence that basal autophagic flux is elevated in aging kidneys (Minami et al. 2023; Yamamoto et al. 2016), which potentially acts as a compensatory mechanism for clearing damaged proteins or protein aggregates. However, autophagy dysfunction has also been reported in aging (Minami et al. 2023), raising the question whether the autophagic process or its molecular machinery remains intact and effective.

Transcription factor EB (TFEB) is a key regulator of cellular autophagy and lysosomal biogenesis. Experimental evidence indicates that TFEB plays a crucial role in multiple stages of autophagy, including initiation, nucleation, elongation, and fusion (Di Malta et al. 2019; Settembre et al. 2011). Upon nuclear translocation, TFEB binds to the promoter regions of autophagy‐related genes—such as beclin1 (BECN1), autophagy‐related 5 (ATG5), sequestosome 1 (SQSTM1/p62), and member RAS oncogene family 7 (RAB7)—to promote their transcription and expression, thereby facilitating autophagy progression (Di Malta et al. 2019). TFEB has been implicated in AKI of various etiologies, including nephrotoxin‐induced AKI (Dong et al. 2024; Wang et al. 2023; Zhu et al. 2020), ischemia–reperfusion AKI (Wang et al. 2019) and septic AKI (Li et al. 2021). Previous studies have reported that autophagy dysregulation in diabetic nephropathy is associated with SMAD family member 3 (SMAD3)‐mediated suppression of TFEB (Yang et al. 2020), and that TFEB expression and functional activity become progressively disrupted during aging (Abokyi et al. 2023; Curnock et al. 2023). However, it is unclear whether autophagy dysfunction in aging kidneys is directly linked to TFEB. Moreover, the role of TFEB in AKI susceptibility in aging kidneys is unknown.

This study was designed to examine age‐related alterations in autophagy during sepsis‐induced AKI. We further proved a critical role of autophagy deficiency in AKI susceptibility in aging kidneys. Mechanistically, we identified TFEB as a key factor responsible for autophagy deficiency in aging kidneys.

## 2 Results

### 2.1 Aging Mice Had Much More Severe AKI After LPS Exposure Than Young Mice

**FIGURE 1:**
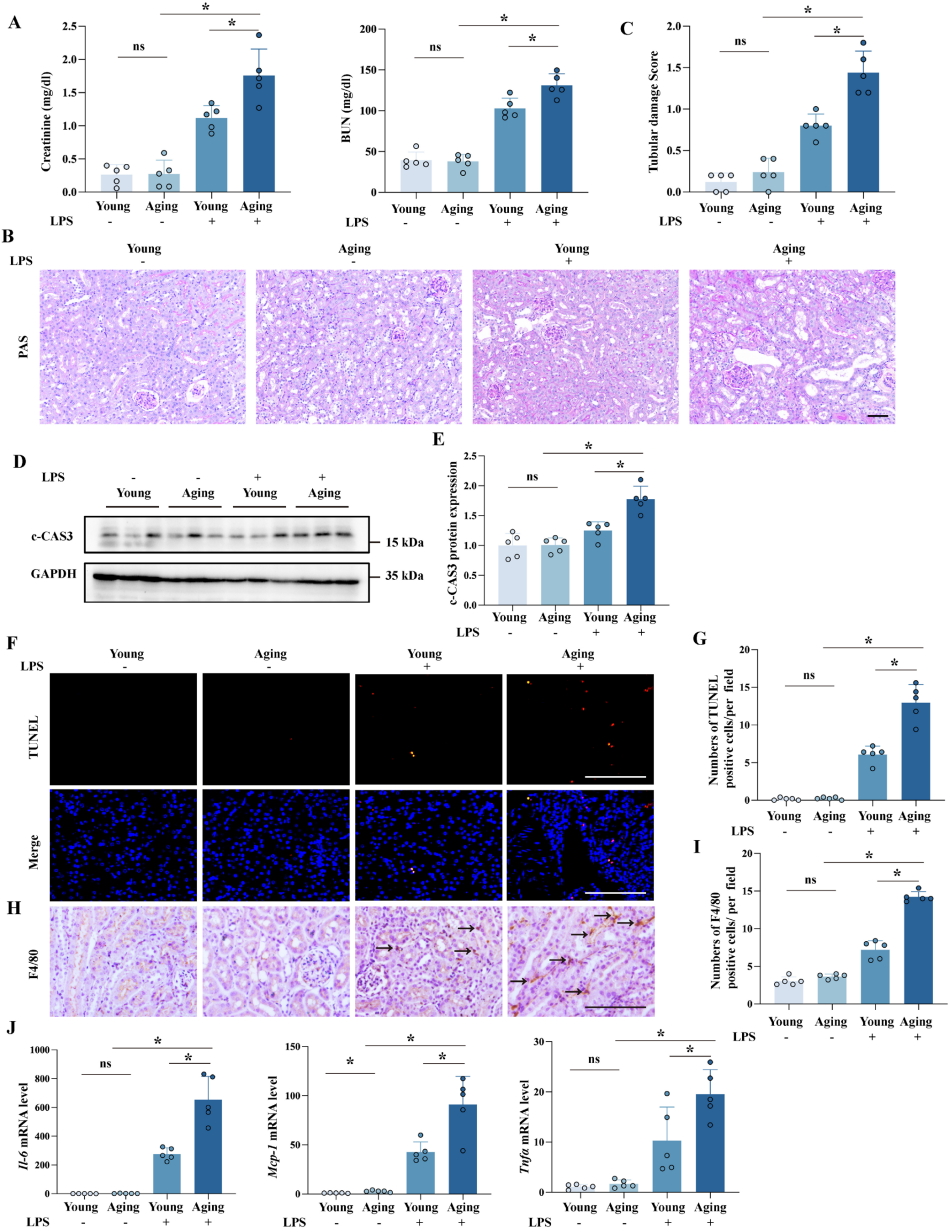
Aging kidneys are more vulnerable to LPS‐induced Septic AKI. (A–J) Aging (18 months) and young (2 months) male C57BL/6 mice were injected with 10 mg/kg LPS (LPS group) or saline vehicle as control (Con group) to collect samples for analysis 12 h later. (A) Serum creatinine (mg/dL) and BUN (mg/dL). (B) Representative images of PAS staining for renal histology, bar scale = 50 μm. (C) Tubular damage score. (D) Representative immunoblots of c‐CAS3 and GAPDH expression in kidney tissues, and (E) corresponding densitometric quantification. (F) Representative images of TUNEL assay (red). Hochest was used to stain the nucleus (blue); scale bar = 50 μm. (G) Number of TUNEL‐positive cells per field. (H) Immunohistochemical staining of the macrophage marker F4/80 (brown). Hematoxylin was used to stain the nucleus (blue); scale bar = 50 μm. (I) Number of F4/80 positive cells per field. (J) Relative mRNA expression levels of the genes encoding *Il‐6, Mcp‐1* and *Tnfα*, assessed by RT‐qPCR. All quantitative data are expressed as mean ± SEM. *p < 0.05.

To investigate the age‐related susceptibility of kidneys to AKI, we administered the same dose of LPS to induce septic AKI in both young (2 months) and aging (18 months) mice. Kidney and serum samples were collected 12 h post‐treatment for renal function assessment and molecular analysis. As shown in Figure 1, serum creatinine and BUN levels (Figure 1A) indicated a significantly greater decline in renal function in aging mice compared to young mice after LPS treatment. PAS staining further supported this observation, as renal tissue staining (Figure 1B,C) revealed more severe structural damage in aging mice. Consistently, immunoblotting analysis of cleaved caspase‐3 (c‐CAS3) (Figure 1D,E) and TUNEL staining (Figure 1F,G) demonstrated markedly increased apoptosis in the kidneys of aging mice in comparison with young mice. Additionally, we assessed the inflammatory response in these animal kidneys. Immunohistochemical staining for F4/80 (Figure 1H,I) revealed more pronounced macrophage infiltration in aging kidneys after LPS treatment. Furthermore, qRT‐PCR analysis of inflammatory markers, including *Il‐6*, *Mcp‐1*, and *Tnfα* (Figure 1J), confirmed an exacerbated inflammatory response in LPS‐treated aging mice kidneys. Together, these tests indicate that LPS induces more severe septic AKI in aging mice than in young mice.

### 2.2 LPS‐Induced Autophagic Activation Was Lower in Aging Kidneys and Senescent Renal Tubular Cells

**FIGURE 2:**
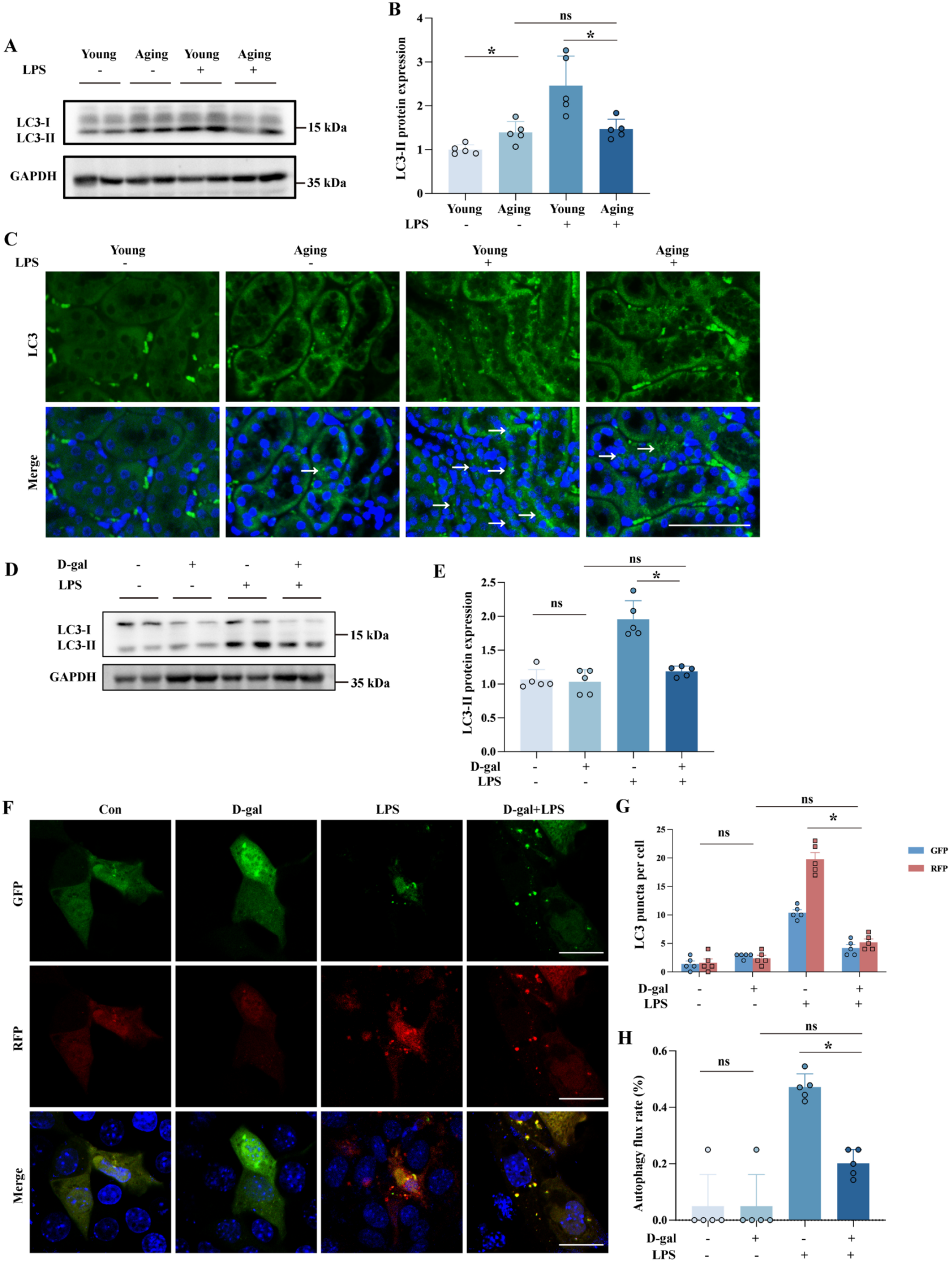
Autophagic activity of renal tubular epithelial cells decreases in the elderly mouse. (A–C) Aging (18 months) and young (2 months) male C57BL/6 mice were injected with 10 mg/kg LPS (LPS group) or saline vehicle as control (Con group) to collect samples for analysis 12 h later. (A) Representative immunoblots of LC3‐I/II and GAPDH expression in kidney tissues, and (B) corresponding densitometric quantification of LC3‐II. (C) Representative images of LC3B immunofluorescence (Green) and the nucleus (Hoechst, blue) in kidney tubules; bar scale = 20 μm. (D–H) BUMPT cells were first cultured in DMEM supplemented with 10% FBS and treated with 300 mM D‐galactose for 72 h to induce cellular senescence (D‐galactose group), while control cells were maintained under identical conditions without D‐galactose (Con group). Following senescence induction, both senescent and control cells were subjected to LPS stimulation. Specifically, cells were incubated in DMEM containing 0.2% FBS and 10 μg/mL LPS for an additional 24 h (D‐galactose + LPS group and LPS group, respectively), whereas corresponding control groups were cultured in the same medium without LPS (D‐galactose group and Con group). (D) Representative immunoblots of LC3‐I/II and GAPDH expression in BUMPT cells, and (E) corresponding densitometric quantification of LC3‐II. (F) BUMPT cells were transiently transfected with mRFP‐GFP‐LC3. RFP and GFP fluorescence images were collected at 24 h after transfection by confocal microscopy; bar scale = 10 μm. (G) The numbers of GFP‐LC3 puncta per cell and RFP‐LC3 puncta per cell were counted separately using ImageJ. The number of autophagosomes is represented by GFP dots, and the number of autolysosomes was obtained by subtracting GFP dots from RFP dots. (H) Autophagic flux rate. Quantitative data are expressed as mean ± SEM. *p < 0.05.

In view of the observation that autophagy is activated as a protective response against in LPS‐induced septic AKI (Mei et al. 2016; Sunahara et al. 2018), we hypothesized that the susceptibility of aging mice to septic AKI might be related to autophagy deficiency. To test this, we initially analyzed LC3 expression in young versus old mice kidneys. Without treatment, old mice kidneys seemed to have a little more LC3‐II than young. But, upon LPS treatment, LC3‐II was significantly lower in old mice kidneys than in young mice kidneys (Figure 2A,B). Consistently, LC3 immunofluorescence showed that LPS stimulation led to a significant accumulation of autophagosomes in young mice kidneys, but not in aging mice (Figure 2C).

In vitro, we established a senescent renal tubular cell model by treating BUMPT cells with D‐gal (Figure S1A–D). D‐gal treatment induced senescence as shown by SA‐β‐gal staining and p53, p21, and p16 expression. Importantly, LPS induced significantly higher levels of apoptosis in senescent cells than in normal/non‐senescent cells as shown by caspase‐3 cleavage and TUNEL staining (Figure S1E–G). We then assessed autophagic activity in these cells. As shown in Figure 2D,E, while LPS induced an obvious increase in LC3‐II expression in normal BUMPT cells, which was mostly absent in senescent cells. To visualize autophagic activity more directly, we transfected normal and senescent BUMPT cells with RFP‐GFP‐LC3 plasmids and subjected them to LPS treatment (Figure 2F–H). Without treatment, the basal autophagic activity was indifferent in senescent cells vs. normal cells. Upon LPS stimulation, normal BUMPT cells exhibited a robust autophagy activation, which was lower in senescent cells. We further isolated primary renal tubular epithelial cells from young and aging mice for in vitro culture (Figure S2A–C). We then tested their response to LPS treatment. In the tubular cells from young mice, LPS induced a significant increase in the expression of LC3‐II, while no or very marginal LC3‐II induction was detected in the tubular cells isolated from aging mice (Figure S2D–E). During LPS treatment, aging tubular cells showed more severe apoptotic and inflammatory responses (Figure S2F–H). We further assessed p62 expression in both in vivo and in vitro models following LPS treatment. As shown in Figure S3A–D, aged kidneys and senescent tubular cells had significantly more p62 after LPS treatment than their young counterparts, suggesting impaired autophagic degradation in the aging kidney.

These in vivo and in vitro analyses indicate that aging kidneys may maintain the basal level of autophagy under normal, non‐challenged conditions but their autophagic response to stress (e.g., sepsis) is significantly lower.

To further assess autophagic flux, we performed lysosomal inhibition assays in BUMPT cells. As shown in Figure S3E, p16‐positive senescent tubules exhibited significantly greater accumulation of LC3‐positive puncta than non‐senescent tubules. Furthermore, Bafilomycin A1 induced marked accumulation of LC3‐II and p62 in control cells but had minimal effects on LC3‐II accumulation in senescent cells, while p62 remained elevated (Figure S3F–I). These findings indicate that the increased LC3‐positive puncta in aged kidneys are primarily attributable to impaired autophagic flux and defective autophagic clearance rather than enhanced autophagic activity, further supporting the presence of autophagy dysfunction in the aging kidney.

### 2.3 Autophagy Regulates LPS‐Induced Injury and Senescence Progression in Senescent BUMPT Cells

**FIGURE 3:**
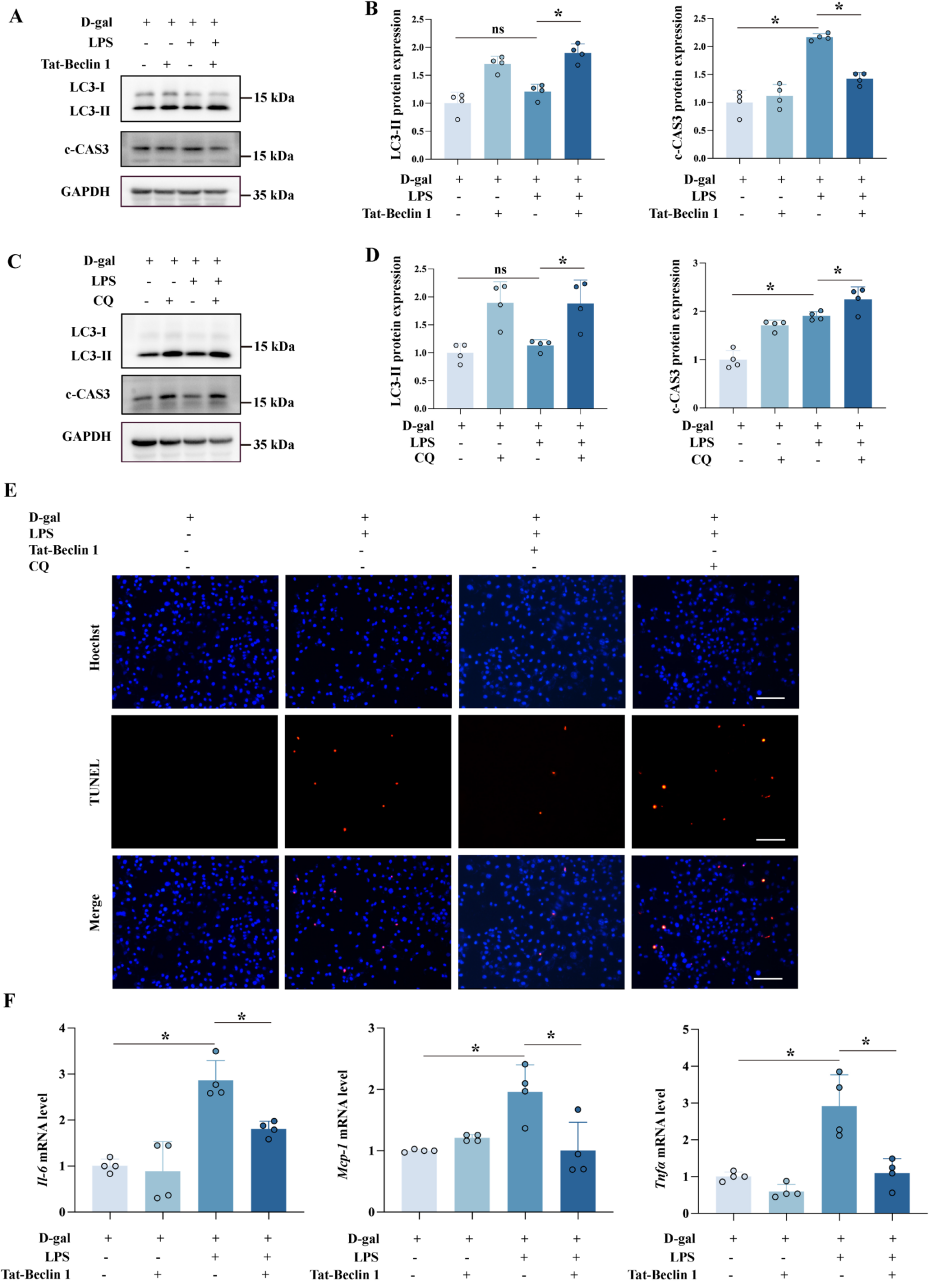
Modulation of autophagy in senescent BUMPT cells bidirectionally regulates their susceptibility to LPS‐induced injury. (A–F) Treatment of LPS‐treated senescent BUMPT cells with Tat‐Beclin 1 peptide (30 μM) and chloroquine (20 μM), respectively. (A) Representative immunoblots of LC3‐I/II, c‐CAS3 and GAPDH expression of BUMPT cells treated with Tat‐Beclin 1 peptide, and (B) corresponding densitometric quantification. (C) Representative immunoblots of LC3‐I/II, c‐CAS3 and GAPDH expression of BUMPT cells treated with chloroquine and (D) corresponding densitometric quantification. (E) Representative images of TUNEL assay (red). Hochest was used to stain the nucleus (blue); scale bar = 50 μm. (F) Relative mRNA expression levels of the genes encoding *Il‐6, Mcp‐1* and *Tnfα*, assessed by RT‐qPCR. All quantitative data are expressed as mean ± SEM. *p < 0.05.

We then determined whether autophagy modulation may influence the response of senescent renal proximal tubular cells to LPS. Specifically, we tested the effects of the autophagy activator Tat‐Beclin 1 peptide or autophagy inhibitor chloroquine (CQ) in senescent BUMPT cells (Figure 3A–D): Tat‐Beclin 1 markedly reduced LPS‐induced c‐CAS3 expression, whereas CQ had the opposite effect. Consistently, TUNEL staining confirmed that Tat‐Beclin 1 reduced, whereas CQ increased LPS‐induced apoptosis in senescent BUMPT cells (Figure 3E). Moreover, induction of autophagy with Tat‐Beclin 1 also attenuated LPS‐triggered inflammatory responses (Figure 3F). Collectively, these results demonstrate a critical role of autophagy deficiency in the susceptibility of senescent renal proximal tubular cells to LPS, highlighting autophagy as a key mediator of cell survival and death under septic stress.

Subsequently, we investigated whether the increased apoptosis induced by autophagy inhibition could reduce the overall senescence burden and improve LPS‐induced injury. To address this, we assessed the effects of CQ‐mediated autophagy inhibition on senescence (SA‐β‐gal activity), cell viability, and inflammatory cytokine expression (Figure S4). Although CQ increased apoptosis in senescent BUMPT cells, it did not reduce the senescence burden. Instead, CQ further increased p16, p21, and p53 expression, SA‐β‐gal positivity, inflammatory cytokine production, and reduced cell viability (Figure S4A–E). Moreover, autophagy inhibition did not improve LPS‐induced injury. These findings suggest that CQ‐induced apoptosis does not act as a beneficial senolytic mechanism. Rather, autophagy inhibition exacerbates cellular stress and injury, leading to increased senescence‐associated phenotypes. Furthermore, time‐course studies demonstrated that both autophagy and senescence markers were induced following LPS treatment, reaching peak levels at 24 h in normal BUMPTs (Figure S5A,B). Subsequent autophagy modulation showed that activation of autophagy reduced the senescence burden, whereas autophagy inhibition further increased it (Figure S5C–F). Collectively, these results suggest that autophagy plays a protective role under septic stress by limiting cellular senescence, while impaired autophagy promotes senescence.

### 2.4 snRNA‐Seq Revealed Differentially Expressed Autophagy Genes Between Young and Aging Mice Kidneys Following LPS Treatment

**FIGURE 4:**
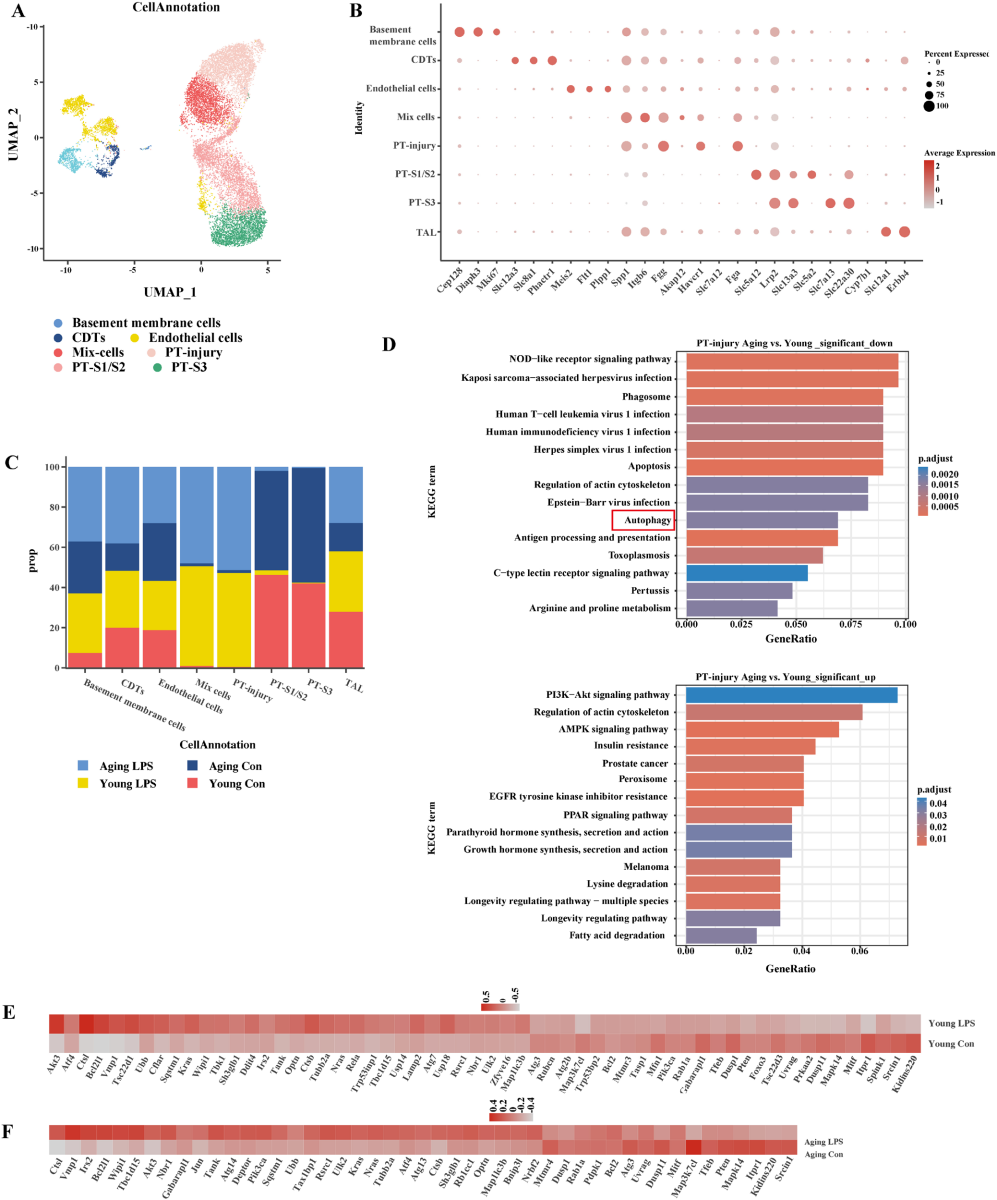
Changes of autophagy signaling pathway genes in single‐nucleus sequencing (snRNA‐seq) between young/aging mice control group and LPS group in kidney tissues. (A–D) Young (2 months) and aging (18 months) mice were intraperitoneally injected with saline and 10 mg/kg LPS, and the renal cortex was collected for snRNA‐seq analysis 12 h later. (A) The Uniform Manifold Approximation and Projection (UMAP) plot showing renal tubular principal cell subpopulations. (B) Cell clusters were identified by kidney cell lineage‐specific marker expression. (C) Bar plot showing the compositions of cell clusters in young/aging control and LPS kidneys. (D) Histogram of differences in cell signaling pathways between PT‐injury cells and PT‐S1/S2/S3 cells based on the KEGG database. (E) Differential genes of autophagy pathway in PT cells of Con group versus LPS group in young mouse based on KEGG database. (F) Differential genes of autophagy pathway in PT cells of Con group vs. LPS group in aging mouse based on KEGG database. PC, principal cell; PT‐injury, proximal tubule cells expressing kidney injury marker genes; PT‐S1, the S1 segment of proximal tubule; PT‐S2, the S2 segment of proximal tubule; PT‐S3, the S3 segment of proximal tubule; TAL, thick ascending limb of the loop of Henle; CDTs, collecting duct cells.

To investigate the underlying mechanisms of autophagy alterations in renal tubular cells of aging mice, we performed single cell sequencing (snRNA‐seq) on renal cortex samples collected from control or LPS‐treated young and aging mice. Figure 4A presents a UMAP plot of main renal tubular cell subpopulations, with clustering defined by lineage‐specific marker genes (Figure 4B). Figure 4C depicts the distribution of these clusters across different intervention groups. Gene Set Enrichment Analysis (GSEA) was performed on injured renal tubular cells from young and aged kidneys using the KEGG database. The analysis revealed significant downregulation of autophagy‐related pathways in aged kidneys (Figure 4D), implicating impaired autophagy as a potential contributor to the exacerbation of LPS‐induced AKI in aging kidneys. To further explore the mechanism underlying autophagic defects in aging kidneys, differentially expressed autophagy‐related genes between young and aged groups were identified (Figure 4E,F).

### 2.5 The Expression of Transcription Factor TFEB in Aged Renal Tubular Epithelial Cells Decreased More Significantly After LPS Induction

**FIGURE 5:**
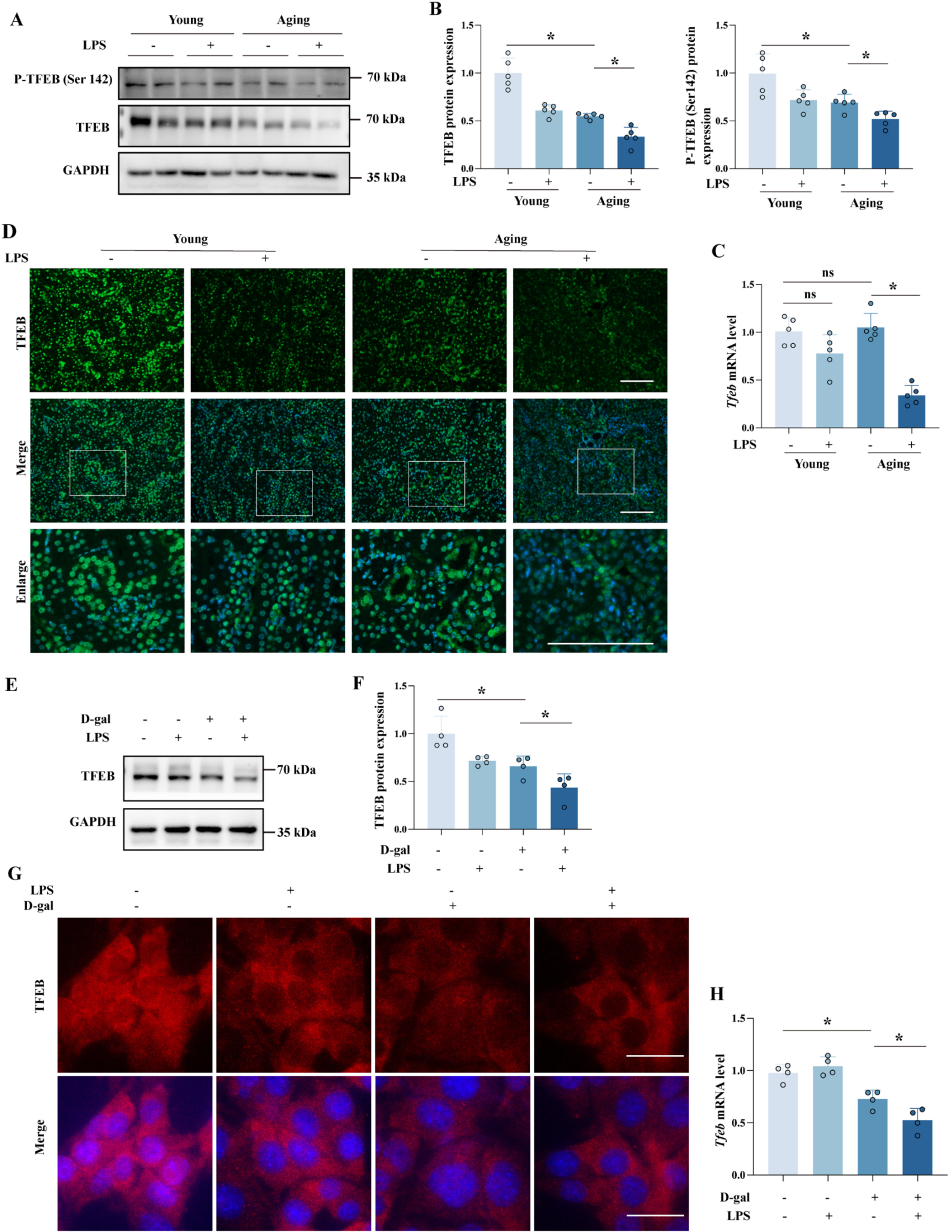
The expression of transcription factor TFEB in aged renal tubular epithelial cells decreased more significantly after LPS induction. (A–D) Aging (18 months) and young (2 months) male C57BL/6 mice were injected with 10 mg/kg LPS (LPS group) or saline vehicle as control (Con group) to collect samples for analysis 12 h later. (A) Representative immunoblots of TFEB, P‐TFEB and GAPDH expression in kidney tissues, and (B) corresponding densitometric quantification. (C) Relative mRNA expression levels of the genes encoding *Tfeb* assessed by qRT‐qPCR in kidney tissue. (D) Representative images of TFEB immunofluorescence (Green) and the nucleus (Hoechst, blue) in kidney tubules; bar scale = 50 μm. (E‐H) BUMPT cells were exposed to DMEM medium containing 10% FBS and 300 mM D‐gal for an additional 72 h (D‐gal group) or without D‐gal exposure (Con group). For LPS treatment, induced senescence cells/normal cells were exposed to DMEM medium containing 0.2% FBS and 10 μg/mL LPS for an additional 24 h (D‐gal + LPS group/LPS group) or without LPS exposure (D‐gal group/Con group). (E) Representative immunoblots of TFEB and GAPDH expression in BUMPT cells, and (F) corresponding densitometric quantification. (G)Representative TFEB immunofluorescence counterstained with Hoechst staining of the nucleus in laser‐scanning confocal microscopy; scale bar = 20 μm. (H) Relative mRNA expression levels of the genes encoding *Tfeb* assessed by RT‐qPCR in BUMPT cells. Quantitative data are expressed as mean ± SEM. *p < 0.05.

After identifying differentially expressed genes from the snRNA‐seq analysis, we analyzed the corresponding protein expression levels and selected key candidates based on their expression patterns and functional relevance. Notably, the analysis suggested TFEB, a critical transcription factor of autophagy (Settembre et al. 2011). We then examined TFEB expression in young and aging mice kidneys after LPS treatment. Immunoblotting analysis revealed an age‐dependent reduction in TFEB protein levels, which was further exacerbated by LPS treatment, particularly in the kidneys of aged mice. Protein phosphorylation plays a critical role in the sub‐cellular localization and transcriptional activity of TFEB. Especially, the phosphorylation of TFEB at Ser142 prevents its nuclear translocation and subsequent transcriptional activity (Napolitano et al. 2018). Aging kidneys appeared to have lower levels of P‐TFEB (Ser142) than young kidneys and LPS treatment further reduced P‐TFEB (Figure 5A,B). This decrease in P‐TFEB was likely attributable to the overall decrease in TFEB protein abundance. At the transcriptional level, aging and young mice kidneys had comparable levels of TFEB mRNA expression; however, LPS treatment attenuated TFEB mRNA in aging kidneys but not in young mice kidneys (Figure 5C). These findings suggest that age‐related decrease in TFEB expression may contribute to the decline in autophagic activity. We further performed immunofluorescence analysis (Figure 5D). The results demonstrated a marked reduction in TFEB expression in young mice kidneys following LPS treatment, with an even more pronounced decrease observed in aging mice, particularly in the nucleus. In vitro in cultured BUMPT cells, we confirmed a significant reduction in TFEB expression in D‐gal‐induced‐senescent cells by immunoblotting (Figure 5E,F) and qRT‐PCR analysis (Figure 5H). Immunofluorescence analysis further revealed that LPS treatment led to a decline in TFEB levels, which was more pronounced in senescent BUMPT cells (Figure 5G), especially in the cell nucleus. In conclusion, our findings indicate that LPS induces a more pronounced decrease in TFEB expression in senescent cells, which may underlie the impaired autophagic activation observed in aging cells.

### 2.6 Overexpression of TFEB Partially Restored Autophagic Activity in Senescent BUMPT Cells

**FIGURE 6:**
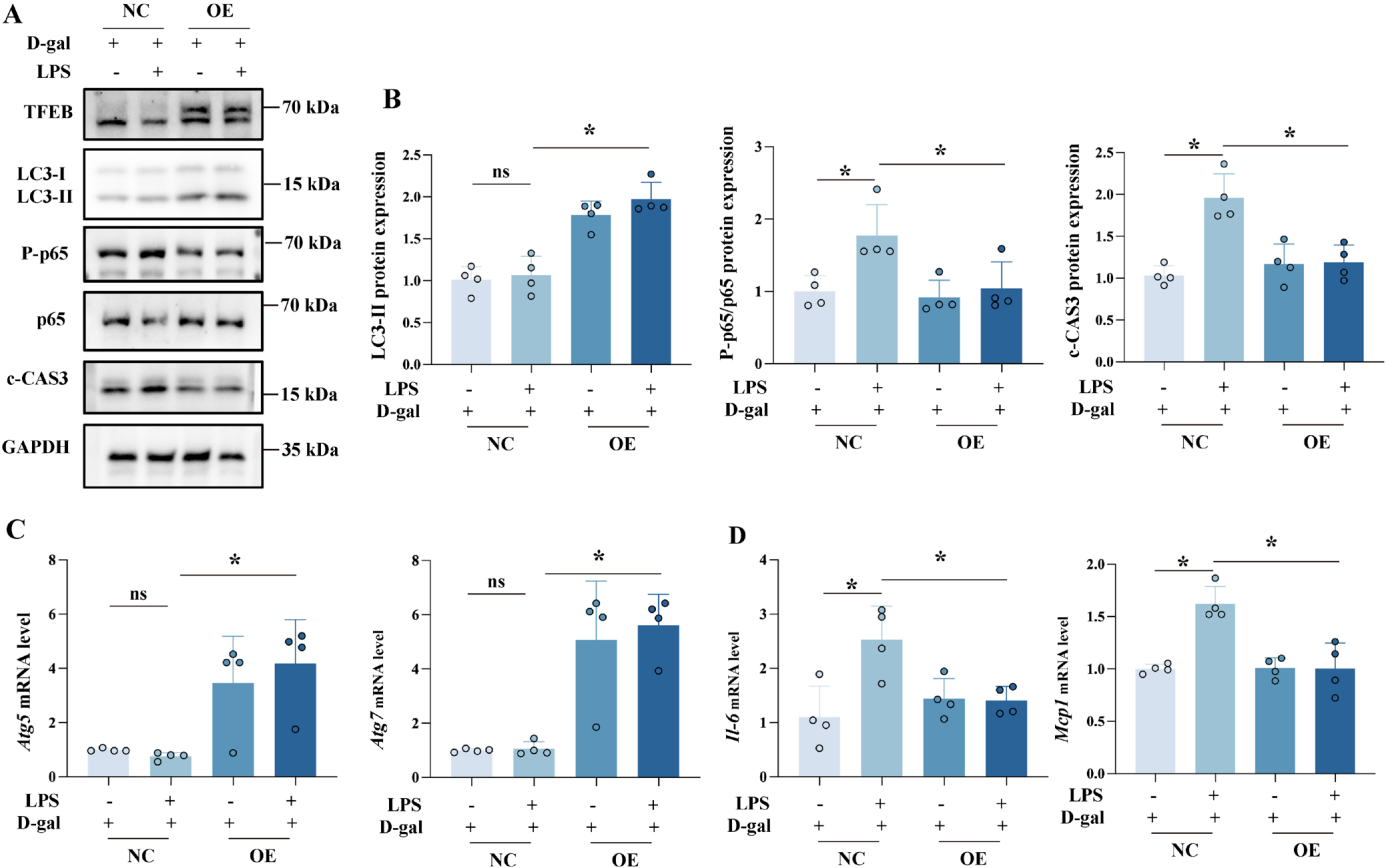
Overexpression of TFEB partially restored autophagy by promoting the transcription of ATG5 and ATG7 in induced senescence BUMPT cells in vitro. (A–D) BUMPT cells were exposed to DMEM medium containing 10% FBS and 300 mM D‐gal for an additional 72 h. Cells were then transfected with TFEB‐overexpression‐plasmid (OE group) and empty‐plasmid (NC group) respectively. Cells were collected after 24 h of LPS (10 μg/mL) treatment. (A) Representative immunoblots of TFEB, LC3I/II, p65, P‐p65, c‐CAS3 and GAPDH expression in BUMPT cells, and (B) corresponding densitometric quantification. (C) Relative mRNA expression levels of the genes encoding *Atg5* and *Atg7*, assessed by RT‐qPCR. (D) Relative mRNA expression levels of the genes encoding *Il‐6* and *Mcp‐1*, assessed by RT‐qPCR. All quantitative data are expressed as mean ± SEM. *p < 0.05.

To investigate whether the downregulation of TFEB in aging renal tubular epithelial cells contributes to their autophagic defect, we first examined the effects of TFEB overexpression. As shown in Figure 6, we transfected a TFEB‐overexpressing plasmid into D‐gal‐induced‐senescent BUMPT cells. Immunoblotting analysis revealed that TFEB overexpression significantly increased LC3‐II levels, indicating enhanced autophagic activity in senescent cells (Figure 6A,B). Importantly, TFEB overexpression suppressed caspase‐3 cleavage and p65 phosphorylation (P‐p65) during LPS treatment (Figure 6A,B), suggesting a potential protective effect against LPS‐induced cellular stress. To further elucidate the mechanism by which TFEB overexpression stimulates autophagy, we examined key autophagy‐related proteins transcriptionally regulated by TFEB. The results showed that TFEB overexpression significantly upregulated *Atg5* and *Atg7* expression (Figure 6C), suggesting that TFEB may restore autophagy activation in senescent cells through transcriptional regulation of autophagy‐related genes. Furthermore, we assessed the expression levels of inflammatory cytokines following TFEB overexpression and found that TFEB overexpression attenuated LPS‐induced *Il‐6* and *Mcp‐1* expression (Figure 6D). These genetic rescue experiments suggest that downregulation of TFEB is a key to autophagy defects and injury susceptibility of senescent kidney tubular cells.

To further evaluate the role of TFEB in cellular senescence, we overexpressed TFEB in D‐galactose‐induced senescent BUMPT cells and assessed the expression of senescence‐associated markers (p16, p21, and p53) (Figure S6). TFEB overexpression markedly reduced the expression of these markers, indicating that restoration of TFEB alleviates cellular senescence and suggesting a protective role for TFEB against aging‐associated cellular dysfunction.

### 2.7 Curcumin Analog C1 Enhanced Autophagy and Protected Aging Kidneys From LPS‐Induced AKI

**FIGURE 7:**
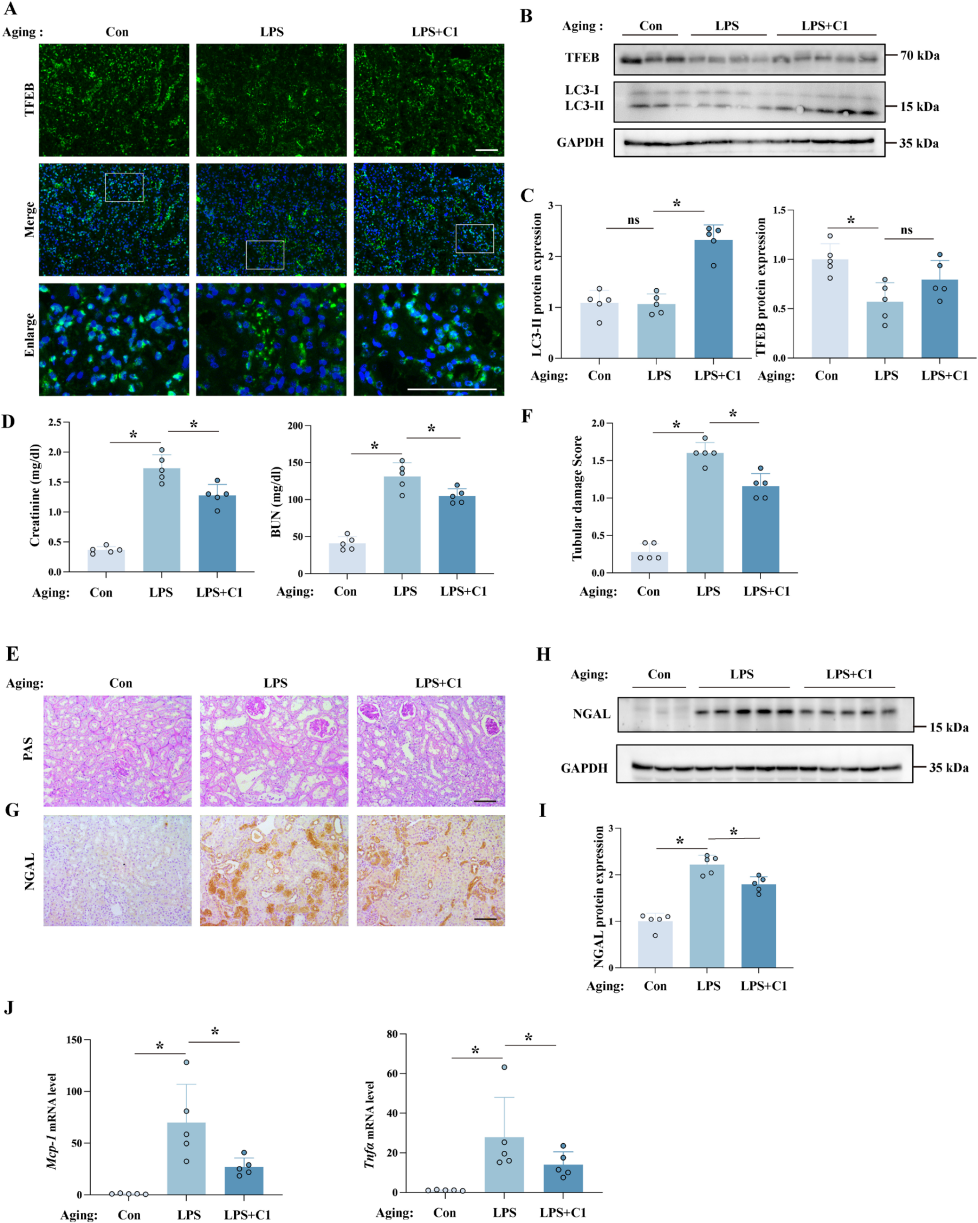
Activation of TFEB protects kidney in aging septic AKI by promoting autophagy. (A–J) Aging (18 months) male C57BL/6 mice were pretreated with curcumin analog C1 (10mg/kg) or vehicle solution 24 h and 2 h before LPS injection or left untreated as control (Con) to collect samples 12 h after LPS injection. (A) Representative images of TFEB immunofluorescence (Green) and the nucleus (Hoechst, blue) in kidney tubules; bar scale = 50 μm. (B) Representative immunoblots of TFEB, LC3I/II, and GAPDH expression in kidney, and (C) corresponding densitometric quantification. (D) Serum creatinine (mg/dL) and BUN (mg/dL). (E) Representative images of PAS staining for renal histology. (F) Tubular damage score. (G) Immunohistochemical staining of the NGAL (brown). Hematoxylin was used to stain the nucleus (blue); scale bar = 50 μm. (H) Representative immunoblots of NGAL and GAPDH expression in kidney tissues, and (I) corresponding densitometric quantification. (J) Relative mRNA expression levels of the genes encoding *Mcp‐1* and *Tnfα*, assessed by RT‐qPCR. All quantitative data are expressed as mean ± SEM. *p < 0.05.

Curcumin analog C1, a specific TFEB activator that promotes its nuclear translocation (Song et al. 2016), was used to assess the role of TFEB in aged renal tubular epithelial cells (Figure S7). C1 treatment increased LC3‐II levels and reduced LPS‐induced cleaved caspase‐3 expression in cells isolated from aging mice (Figure S7A,B). In addition, qRT‐PCR analysis showed that C1 attenuated the LPS‐induced upregulation of *Il‐6, Mcp‐1*, and *Tnfa* (Figure S7C). We further evaluated the in vivo efficacy of C1 in mitigating LPS‐induced septic AKI in aging mice. C1 effectively inhibited LPS‐induced TFEB nuclear export in the kidney (Figure 7A) but did not significantly prevent the LPS‐induced reduction in TFEB expression (Figure 7B,C). This observation is consistent with a previous report (Song et al. 2016) that C1 specifically promotes TFEB nuclear translocation. Moreover, C1 enhanced LC3‐II levels, indicating that TFEB nuclear translocation promotes autophagy activation in aging kidneys. We then evaluated renal function by measuring serum creatinine and blood urea nitrogen (BUN) levels. As shown in Figure 7D, C1 partially but significantly alleviated LPS‐induced renal dysfunction in aging mice. Histological analysis, including tubular injury scoring and PAS staining, further confirmed that C1 ameliorated kidney damage during LPS treatment (Figure 7E,F). Additionally. Immunoblotting and immunohistochemical staining of NGAL (a kidney injury biomarker (Cerda et al. 2025)) revealed that C1 mitigated LPS‐induced AKI in aging kidneys (Figure 7G–I). To examine the inflammatory response, we performed qRT‐PCR and found that C1 treatment reduced LPS‐induced upregulation of *Mcp‐1* and *Tnfa* in aging kidneys (Figure 7J). Taken together, these findings further support the protective role of TFEB activation in LPS‐induced AKI in aging kidneys.

## 3 Discussion

The aging population faces numerous health challenges, among which the prevalence of kidney diseases has risen significantly in the elderly (Duarte et al. 2020). Cellular senescence, characterized by irreversible cell cycle arrest (Gorgoulis et al. 2019; van Deursen 2014), is considered a key pathogenic factor leading to kidney disease (Huang et al. 2022; O'Sullivan et al. 2017). Kidney aging is marked by a decline in renal function, the accumulation of harmful metabolites, and a diminished capacity for defense against various insults (Docherty et al. 2019; Hommos et al. 2017). Therefore, the aging kidney exhibits a markedly increased susceptibility to both AKI and CKD (Muroya et al. 2018; O'Sullivan et al. 2017). Consistently, in our present study, aging mice developed more severe AKI after LPS treatment than young mice. Importantly, we demonstrate that defective autophagy activation is a key to the heightened AKI susceptibility in aging mice. At the molecular level, our work suggests that TFEB is down‐regulated in senescent renal tubule cells, contributing to the lack of autophagic response in the aging kidney. Therapeutically, we show that restoration of TFEB or autophagy may diminish the AKI susceptibility in aging mice.

In the kidney, autophagy plays a crucial protective role against AKI (Li et al. 2025; Tang et al. 2020; Xiang et al. 2023). This protective function is primarily achieved through the degradation of toxic protein aggregates and removal damaged subcellular organelles, thereby maintaining cellular homeostasis (Minami et al. 2023; Xiang et al. 2023). As cells age or become senescent, factors such as telomere dysfunction, the accumulation of DNA damage, and an increasing burden of misfolded proteins may lead to an increase in autophagy activity (Di Micco et al. 2021; Shmulevich and Krizhanovsky 2021). Consistently, in our study the basal level of autophagy in aging or senescent renal tubular epithelial cells is marginally higher than that in young cells (Figure 2A–C). Aging‐related chronic cellular stress, including the accumulation of damaged proteins and dysfunctional organelles, may induce a compensatory increase in basal autophagosome formation. Consistently, we observed increased LC3‐positive puncta in senescent tubular cells (Figure S3E). Of note, elevated LC3 signals do not necessarily indicate enhanced autophagic activity, as autophagosome accumulation can also result from impaired autophagic degradation. Supporting this notion, Bafilomycin A1 induced substantial LC3‐II accumulation in young tubular cells but had minimal effects in senescent cells, indicating impaired autophagic flux (Figure S3F–I). Therefore, the elevated LC3 signal observed in senescent cells may reflect the combined effect of increased basal autophagosome formation and impaired autophagic clearance. However, following LPS treatment, young mouse kidneys or renal tubular epithelial cells can rapidly activate autophagy, whereas this autophagic response to stress was markedly diminished in aging kidneys or cells. This observation suggests an aging‐related decline in cellular “reserve” of autophagy, limiting the adaptive response to injury or pathogenic insults. In vitro, we modulated autophagy in senescent cells using TAT‐Beclin1 peptide or chloroquine, which respectively enhanced or blocked autophagy. The results show that TAT‐Beclin1 alleviated, whereas chloroquine augmented, cell injury and death during LPS treatment (Figure 3), further confirming the critical role of autophagy in the stress response of senescent cells. These findings underscore autophagy modulation as an effective strategy for enhancing the resilience of aging kidneys and senescent cells against AKI.

The decline in autophagic activity may result from multiple factors. In our study, the “exhaustion” of cellular autophagic capacity appears to be linked to aging‐related diseases. To explore the mechanisms underlying autophagy dysregulation in the aging kidney, we performed snRNA‐seq analysis, identifying autophagy‐related genes differentially expressed after septic AKI in aging mice (Figure 4). Based on the RNA‐seq data and the experiments in primary tubular epithelial cells, we found that TFEB, a key transcription factor and regulator of the autophagy‐lysosomal pathway, was significantly downregulated in the aging kidney following LPS treatment (Figure 5A–D). This down‐regulation was accompanied by a marked decrease in nuclear TFEB accumulation (Figure 5D). It is worth noting that most previous studies showed that the mTOR pathway is persistently activated in senescent cells (Weichhart 2017), possibly accompanied by increased phosphorylation of TFEB at the Ser142 site to prevent the nuclear translocation of TFEB. In our study, TFEB phosphorylation at Ser142 was significantly reduced after LPS stimulation in senescent cells, which may be due to the decrease in total TFEB. The precise mechanistic relationship between the mTOR pathway and TFEB in sepsis‐induced AKI in the elderly has yet to be fully elucidated and warrants further investigation.

Our study demonstrates that TFEB protein levels decline with age, whereas basal autophagic activity is elevated in older individuals compared with younger counterparts. This apparent discrepancy likely reflects the complex, multilayered regulation of autophagy, as basal autophagy is strongly modulated by multiple factors, including nutrient availability, cellular energy status, and the activity of autophagy suppressors (Aman et al. 2021; Rubinsztein et al. 2011). Critically, once TFEB expression falls below a functional threshold, the capacity of cells to further activate autophagy in response to stress may be markedly compromised. This impairment in stress‐induced autophagy may represent a key mechanism underlying the heightened susceptibility of aged kidneys to injury. In young kidneys, LPS stimulation can promote autophagy initiation and enhance autophagic flux through stress‐responsive pathways such as AMPK–ULK1 signaling and JNK activation, thereby eliciting a robust and cytoprotective autophagic response (Mei et al. 2016; Zhao et al. 2020). In contrast, TFEB primarily governs lysosomal biogenesis and the transcriptional reprogramming of autophagy‐related genes, processes that are critically dependent on its nuclear translocation and transcriptional activity (Jeong et al. 2021; Settembre et al. 2011). Under inflammatory stress, TFEB function may be further suppressed by mTORC1‐mediated inhibition, enhanced nuclear export, or accelerated protein degradation, resulting in a reduction in total TFEB abundance. Collectively, these alterations may further exacerbate autophagic insufficiency in aged tubular cells exposed to LPS, ultimately amplifying renal vulnerability to inflammatory injury.

Our findings suggest that defective autophagic activation in aging kidneys may be closely related to the downregulation of TFEB, particularly its transcription activity in the cell nucleus. To support this, we overexpressed TFEB in cultured renal tubular cells and activated its nuclear translocation with C1 (Figures 6 and 7). The results demonstrate a significant restoration of autophagy activation with consequent cyto‐protection during LPS treatment. Additionally, TFEB activation by C1 restored the expression of autophagy‐related genes, especially ATG5 and ATG7, which are crucial components of the ubiquitin‐like conjugation system involved in the formation of autophagosome (Changotra et al. 2022; Tang et al. 2020). Specifically, ATG5 and ATG7 facilitate the conversion of cytoplasmic LC3‐I to membrane‐bound LC3‐II, playing a central role in membrane expansion during autophagosome formation (Lőrincz and Juhász 2020; Walczak and Martens 2013). TFEB has been reported to regulate ATG5 and ATG7 expression, either directly or indirectly (Abokyi et al. 2023), and in our study, TFEB activation stimulated the mRNA expression of ATG5 and ATG7, which may contribute to the restoration of autophagic activity. We have further validated the effects of TFEB activation by C1 in vivo in aging mouse kidneys, reinforcing the role of TFEB and autophagy in aging‐associated functional decline and diseases.

In addition to TFEB, there may be other autophagy regulators contributing to the defective autophagic response in aging kidneys. In this regard, our RNA‐seq data revealed a significant downregulation of *Rubcn*, a known negative regulator of autophagy, following LPS treatment. A role of *Rubcn* in suppressing autophagy during aging has been suggested (Nakamura et al. 2019; Yamamuro et al. 2020). Nakamura and colleagues detected an increase of *Rubcn* expression in aged worm, fly, and mouse tissues and, remarkably, knockdown of *Rubcn* extended worm and fly lifespan (Nakamura et al. 2019). Therefore, while our current work pinpoints the down‐regulation of TFEB in the autophagic deficiency in aging kidneys, the work does not exclude the involvement of other autophagy regulators, such as *Rubcn*.

## 4 Conclusion

We have verified the AKI susceptibility of aging kidneys using both in vivo mouse and in vitro tubular cell culture models. We demonstrate that the lack of autophagic response is critical to the AKI susceptibility in aging. Furthermore, we have identified the downregulation of TFEB as a central mediator of autophagic defects in aging kidneys and senescent cells. Pharmacological modulation of TFEB and autophagy may confer renal protection in elderly individuals during sepsis.

## 5 Methods

### 5.1 Animals and Treatment

Animal experiments were approved by the Institutional Animal Care and Use Committee of the Second Xiangya Hospital. C57BL/6 mice were obtained from SJA Laboratory Animal Corporation (Hunan, China). Young mice were about 8 weeks old, and old mice were about 18–20 months old. Septic AKI was induced by lipopolysaccharides (LPS) treatment (Sigma, L2880) at 10 mg/kg. For curcumin analog C1 treatment (C1, [Selleck, S6769]), it was injected at 10 mg/kg intraperitoneally 24 h and 2 h before LPS treatment. Mice were killed 12 h post LPS treatment, and kidneys were either snap‐frozen in liquid nitrogen or fixed in 10% neutral buffered formalin. Five animals were included in each experimental group. All animal research was approved by the Institutional Animal Care and Use Committee of the Second Xiangya Hospital, Central South University (Approval No. 2022418). And all animal procedures conformed to the NIH Guide for the Care and Use of Laboratory Animals.

### 5.2 Cell Culture and Treatment

The Boston University mouse proximal tubular cell line (BUMPT), initially provided by Dr. Lieberthal (Boston, MA), was cultured in DMEM medium supplemented with 10% fetal bovine serum (FBS). To induce senescence, BUMPT cells were incubated with DMEM medium containing 10% FBS and 300 mM D‐ Galactose (Sigma‐Aldrich, G0750) for 72 h (Miao et al. 2019). For LPS treatment, cells were incubated with DMEM medium containing 0.2% FBS and 10 μg/mL LPS for 24 h. For Tat‐Beclin 1 peptide (HY‐P2260, Medchemexpress) treatment, cells were incubated with DMEM medium containing 0.2% FBS and 30 μM Tat‐Beclin 1 peptide for 8 h. For chloroquine (C6628, Sigma‐aldrich) treatment, cells were incubated with DMEM medium containing 0.2% FBS and 20 μM for an additional 8 h. For detection of autophagic flux in cells, normal BUMPT cells and senescence‐induced BUMPT cells were transiently transfected with mRFP‐GFP‐LC3 (ptfLC3, 21,074, Addgene). After treatment, RFP and GFP fluorescence images were collected by confocal microscopy (Zeiss, United States). The numbers of GFP‐LC3 puncta per cell and RFP‐LC3 puncta per cell were counted separately using Image J. The number of autophagosomes was represented by GFP dots, and the number of autolysosomes was obtained by subtracting GFP dots from RFP dots. The number of autolysosomes was divided by the number of RFP puncta to calculate the autophagic flux rate. For overexpression of TFEB, BUMPT cells were transfected with a TFEB overexpression plasmid (pSLenti‐EF1‐EGFP‐P2A‐Puro‐CMV‐Tfeb‐3xFLAG‐WPRE, OBiO Technology) or a control plasmid (pSLenti‐EF1‐EGFP‐P2A‐Puro‐CMV‐MCS‐3xFLAG‐WPRE, OBiO Technology). The in vitro experiments were repeated at least 4 times for statistical analysis.

### 5.3 Isolation of Primary Renal Tubular Epithelial Cells and Treatment

Murine primary tubular epithelial cells (mPTECs) were isolated from above described young and aging mice as previously described (Livingston et al. 2023). In brief, the kidney was collected and minced for digestion with a collagenase/dispase suspension. The digested tissue was passed through a 100 and 70 μm mesh. Subsequently, the cells caught with a 40 μm mesh were isolated and grown in DMEM/F12 (1:1) with 10% FBS at 37°C and 5% CO_2_ in a humidified atmosphere. For LPS treatment, mPTECs were exposed to DMEM medium containing 10% FBS and 10 μg/mL LPS for 24 h. For activation of TFEB, cells were incubated with curcumin analog C1 (5 μM) in medium for 24 h.

### 5.4 Assessment of Serum Creatinine and Blood Urea Nitrogen (BUN)

Serum was obtained from blood samples following clotting and centrifugation. Renal dysfunction was assessed by measuring serum creatinine and blood urea nitrogen (BUN) using reagents from BioAssay Systems (Hayward, CA), as detailed in previous studies (Jiang et al. 2012).

### 5.5 Periodic Acid‐Schiff (PAS) Staining of Kidney Tissues

The staining solution of PAS was purchased from Servicebio Company. After deparaffinization and hydration, the sections were stained with 0.5% periodic acid solution for 10 min and Schiff reagent in the dark for 30 min, followed by counterstaining with hematoxylin. The ‘Tubular Injury Score’ was assigned based on the following criteria: a score of 0 for no apparent damage; 1 for damage involving < 25% of tubules; 2 for damage involving 25%–50% of tubules; 3 for damage involving 50%–75% of tubules; and 4 for damage involving > 75% of tubules. This scoring method has been used in previous studies (Jiang et al. 2012).

### 5.6 Immunoblot Analysis

Renal cortical and outer medulla tissues were lysed using a 2% SDS buffer containing 1% protease inhibitor cocktail (P8340, Sigma‐Aldrich). Protein concentration was determined using a Pierce BCA protein assay kit (23225, Thermo Fisher Scientific). Equal protein amounts were separated through SDS‐polyacrylamide gels and subsequently transferred onto polyvinylidene difluoride membranes. After blocking with 5% skim milk for 1 h, the membrane was incubated with primary antibodies at 4°C overnight and secondary antibodies for 1 h at room temperature. Primary antibodies utilized in the present study were from the following sources: anti‐LC3A/B (9139, Cell Signaling Technology), anti‐TFEB (A303‐673A, Bethyl), anti‐p‐TFEB(Ser142) (bs‐22337R, Bioss), anti‐NGAL (AF1857, R&D Systems), anti‐cleaved caspase‐3 (Asp175) (9661, Cell Signaling Technology), anti‐NF‐kB p65 (8242, Cell Signaling Technology), anti‐phospho‐NF‐kB p65 (3033, Cell Signaling Technology), anti‐P53 (60225‐1‐Ig, Proteintech), anti‐P21 (37543, Cell Signaling Technology), anti‐P16 (29271, Cell Signaling Technology), anti‐GAPDH (10494‐1‐AP, Proteintech). All secondary antibodies for immunoblot analysis were acquired from Thermo Fisher Scientific. Quantification of protein bands was conducted using ImageJ software.

### 5.7 Immunohistochemical Staining

Paraffin‐embedded kidney tissues were cut into 4 μm‐thick sections followed by deparaffinization, hydration, and antigen retrieval via incubation with 0.1 M sodium citrate (pH 6.0) at 100°C. After exposure to 3% H_2_O_2_ and 5% normal goat serum for minimizing non‐specific binding, tissue sections were subjected to anti‐NGAL antibody (AF1857, R&D Systems) and anti‐F4/80 antibody (GB113373, Servicebio) at 4°C overnight. After washing, the sections were incubated with a secondary antibody for 30 min, and the signals were developed using a DAB kit, followed by counterstaining with hematoxylin. For quantification, 10 fields were randomly chosen from each tissue section for counting.

### 5.8 Immunofluorescence Staining

Paraffin‐embedded kidney tissue sections of 4 μm thickness were deparaffinized and incubated with EDTA for antigen retrieval. For LC3B staining, the following steps were performed using Tyramide SuperBoost Kits with Alexa Fluor Tyramides (Thermo Fisher Scientific, Waltham, Massachusetts, USA). The sections were incubated with 3% Hydrogen Peroxide Solution at room temperature for 1 h. After washing with PBS, kidney tissues were blocked for 1 h with blocking buffer. Then the tissues were exposed to 1:5000 anti‐LC3 (NB100‐2220, Novus Biologicals) at 4°C overnight and incubated with poly‐HRP‐conjugated secondary antibody and goat anti‐mouse IgG conjugated with Alexa Fluor 488 for 1 h at room temperature. After washing with PBS, the slides were incubated with a tyramide working solution and Reaction Stop Reagent for 2.5 m each at room temperature. For TFEB staining in kidney tissues, tissue sections were treated with 3% H_2_O_2_, 5% normal goat serum and 0.1% Triton X‐100 to minimize nonspecific binding. Next, the sections were exposed to (1:500) anti‐TFEB (13372‐1‐AP, Proteintech) antibody overnight at 4°C. Following washing, the sections were incubated with corresponding secondary antibody with Alexa Fluor 488 for 1 h at room temperature, with Hoechst serving as a nuclear counterstain. For TFEB staining in BUMPT cells, the cells were washed with phosphate‐buffered saline (PBS) and fixed with cold methanol: acetone (1:1) at room temperature for 10 min. After washing with PBS, fixed cells were blocked for 30 m with 3% normal goat serum. Then the cells were incubated with anti‐TFEB (13372‐1‐AP, Proteintech) overnight at 4°C and exposed to goat anti‐rabbit IgG conjugated with Alexa Fluor 594 for 1 h at room temperature. Hoechst was used as a nuclear counterstain. Images were collected by confocal microscopy (Zeiss, United States).

### 5.9 Single Nucleus RNA Sequencing

SnRNA‐seq was conducted according to our previous study with modifications (Ma et al. 2022). Briefly, cell nuclei were isolated from kidney tissues using a Nucleus Isolation Kit (SHBIO, 52009‐10). The kidneys were cut and transferred into a 5 mL tube containing lysis buffer with RNase inhibitors (Sigma, 3335399001), mixed and lysed for 3 min on ice, then filtered through a 40 μm cell filter (Sigma, BAH136800040). Nucleus count and viability were estimated using Fluorescence Cell Analyzer (Countstar Rigel S2) or SeekMate Tinitan Fluorescence Cell Counter (SeekGene M002C) with AO/PI reagent. After staining with 0.4% trypan blue (Sangon Biotech, E607320‐0001), the nucleus was also observed under a microscope (Jiangnan Novel Optics, XD‐202) to verify the quality. snRNA‐seq libraries were prepared using SeekOne Digital Droplet Single Cell 3′ library preparation kit (SeekGene, Catalog No. K00202). Briefly, an appropriate number of cell nuclei were mixed with reverse transcription reagent and then added to the sample well in SeekOne DD Chip S3. Subsequently, Barcoded Hydrogel Beads (BHBs) and partitioning oil were dispensed into corresponding wells. After emulsion, droplet generation reverse transcription was performed at 42°C for 90 min and inactivated at 85°C for 5 min. Next, cDNA was purified and amplified in PCR reaction. The amplified cDNA product was then cleaned, fragmented, end repaired, A‐tailed and ligated to sequencing adaptor. Finally, the indexed PCR was performed to amplify the DNA representing 3′ polyA part of expressing genes which also contained Cell Barcode and Unique Molecular Index. The indexed sequencing libraries were cleaned up with VAHTS DNA Clean Beads (Vazyme, N411‐01), analyzed by Qubit (Thermo Fisher Scientific, Q33226) and Bio‐Fragment Analyzer (Bioptic, Qsep400). The libraries were then sequenced on illumina NovaSeq 6000 with PE150 read length or DNBSEQ‐T7 platform with PE150 read length.

### 5.10 Quantitative RT‐PCR

Total RNA from kidney tissues or cells was extracted using TRIzol reagents from CWBIO (Jiangsu, China), following the manufacturer's protocol. cDNA synthesis was carried out using Taqman RT reagents (TaKaRa, Japan). Quantitative real‐time PCR was conducted using the TB Green Premix Ex Taq II reagent (TaKaRa) on a LightCycler96 Real‐Time PCR System. The relative expression was normalized to the levels of GAPDH. The sequences of qPCR primers are provided in the Table 1.

**TABLE 1:** Primer sequences used for qPCR in this study.

Primer	Forward	Reverse
*Il‐6*	TCTATACCACTTCACAGTCGGA	GAATTGCCATTGCACAACTCTTT
*Mcp‐1*	TAAAAACCTGGATCGGAACCAAA	GCATTAGCTTCAGATTTACGGGT
*Tnfα*	GCGACGTGGAACTGGCAGAAG	GCCACAAGCAGGAATGAGAAGAG
*Gapdh*	AGGTCGGTGTGAACGGATTTG	GGGGTCGTTGATGGCAACA
*Tfeb*	GCAGCCACCTGAACGTGT A	TGTTAGCTCTCGCTTCTGAGT
*p21*	GACCTGGGAGGGGACAAGAG	TTCTCTTGCAGAAGACCAATC
*P16*	GGCCAATCCCAAGAGCAGAG	GCCACATGCTAGACACGCTA
*Atg7*	TCTGGGAAGCCATAAAGTCAGG	GCGAAGGTCAGGAGCAGAA
*Atg5*	TGTGCTTCGAGATGTGTGGTT	GTCAAATAGCTGACTCTTGGCAA

### 5.11 Apoptosis Analysis

Apoptosis in cultured proximal tubular cells was assessed through a TUNEL assay, employing the In Situ Cell Death Detection Kit from Roche Applied Science (Indianapolis, IN) as described previously (Wei et al. 2018). Positive staining was visualized by fluorescence microscopy. For quantification, 10 fields were randomly chosen from each tissue section and the quantification of TUNEL‐positive cells per microscope area was conducted.

### 5.12 Senescence‐Associated Galactosidase Beta 1 (SA‐β‐Gal) Staining

Tubular senescent change was evaluated through SA‐β‐gal staining of cells with a senescence detection kit (ab65351, Abcam) as recently described (Li et al. 2023). Briefly, cells were cultured on coverslips in 35 mm culture dishes. After treatment, cells were fixed with fixative solution and then incubated with a mixture of staining solutions at 37°C overnight. Then the staining solution was removed, and the dishes were washed with pure water and mounted with glycerol.

### 5.13 Statistical Analyses

GraphPad Prism 9 was used for statistical analysis. Student's *t*‐test was applied to evaluate the differences between two groups. One‐way ANOVA was used to measure the differences among more than two groups. Quantitative data were expressed as mean ± SEM and *p* < 0.05 was considered significantly different.

## Author Contributions

Z.D., D.Z. and Y.X. contributed to the study conception. Methodological design involved Y.X., Y.F., Z.L., W.W., J.C. Experimental work was conducted by Y.X., Y.F., and Y.H. Y.X., Z.L., and Y.F. carried out the data analyses. Supervision of the study was undertaken by Z.D. and D.Z. The manuscript was initially drafted by Y.X. and further revised with input from Z.D. and D.Z. All authors reviewed and approved the final manuscript.

## Funding

The work was supported partly by National Natural Science Foundation of China (No. U25A2034, 82500842, and 82270734).

## Conflicts of Interest

The authors declare no conflicts of interest.

## Supporting information


**Figure S1:** Induced‐senescence BUMPT cells in vitro are more susceptible to LPS treatment.
**Figure S2:** Primary renal tubular cells of old mice cultured in vitro are more sensitive to LPS and have dysregulated autophagy than young mice.
**Figure S3:** Autophagy dysfunction in senescent renal tubular epithelial cells.
**Figure S4:** Autophagy inhibition aggravates LPS‐induced injury and senescence burden in senescent BUMPT cells.
**Figure S5:** Autophagy modulation influences the senescence phenotype of LPS‐treated BUMPT cells.
**Figure S6:** Overexpression of TFEB alleviates D‐gal‐induced senescence in BUMPT cells.
**Figure S7:** Promoting the nuclear translocation of TFEB can protect LPS‐induced apoptosis and inflammation by activating autophagy in mPTECs in vivo.

## Data Availability

Relevant datasets can be obtained from the corresponding authors upon reasonable request.
