# Supplementary material for: Down‐Regulation of TFEB With Defective Autophagy in the Susceptibility of Aging Kidneys to Septic Acute Kidney Injury
Source: Aging Cell. 2026 Jul 21;25(8):e70644. doi: 10.1111/acel.70644 (PMC13387737; doi:10.1111/acel.70644)
Supplement: Supplementary file 1 — Figure S1: Induced‐senescence BUMPT cells in vitro are more susceptible to LPS treatment. Figure S2: Primary renal tubular cells of old mice cultured in vitro are more sensitive to LPS and have dysregulated autophagy than young mice. Figure S3: Autophagy dysfunction in senescent renal tubular epithelial cells. Figure S4: Autophagy inhibition aggravates LPS‐induced injury and senescence burden in senescent BUMPT cells. Figure S5: Autophagy modulation influences the senescence phenotype of LPS‐treated BUMPT cells. Figure S6: Overexpression of TFEB alleviates D‐gal‐induced senescence in BUMPT cells. Figure S7: Promoting the nuclear translocation of TFEB can protect LPS‐induced apoptosis and inflammation by activating autophagy in mPTECs in vivo. [file ACEL-25-e70644-s001.docx]

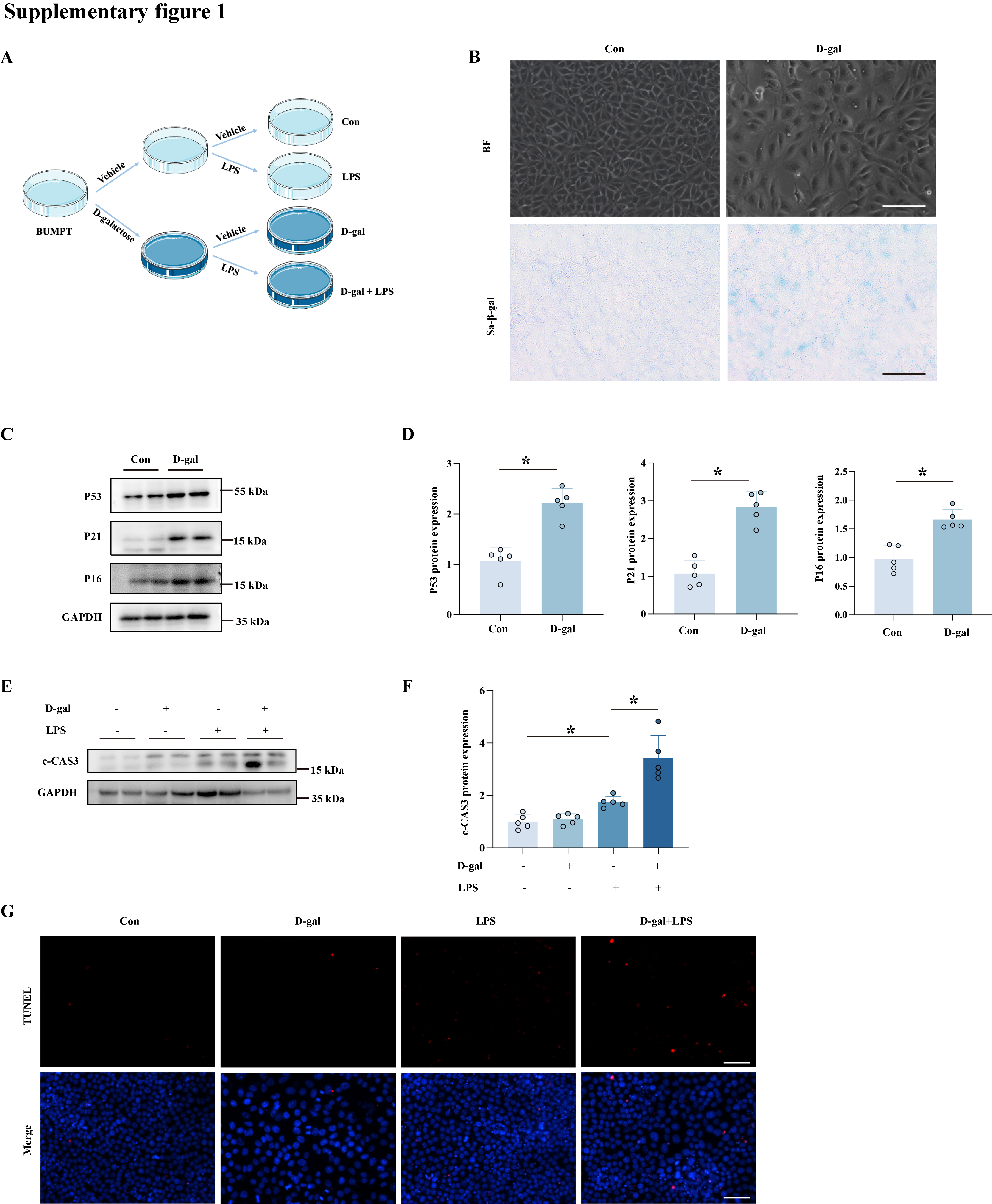


**Supplementary Figure 1. Induced-senescence BUMPT** **cells in vitro are more susceptible to LPS treatment.**

(A-G) BUMPT cells were exposed to DMEM medium containing 10% FBS and 300mM D-gal for an additional 72 hours (D-gal group) or without D-gal exposure (Con group). For LPS treatment, induced senescence cells / normal cells were exposed to DMEM medium containing 0.2% FBS and 10μg/ml LPS for an additional 24 hours (D-gal + LPS group / LPS group) or without LPS exposure (D-gal group / Con group). (A) BUMPT cell intervention flow chart. (B) Representative images of SA-b-gal staining in BUMPT cells. (C) Representative immunoblots of P53, P21, P16 and GAPDH expression in BUMPT cells, and (D) corresponding densitometric quantification. (E) Representative immunoblots of c-CAS3 and GAPDH expression in BUMPT cells, and (F) corresponding densitometric quantification. (G) Representative images of TUNEL assay (red) in BUMPT cells. Hochest was used to stain the nucleus (blue). All quantitative data are expressed as mean ± SEM.
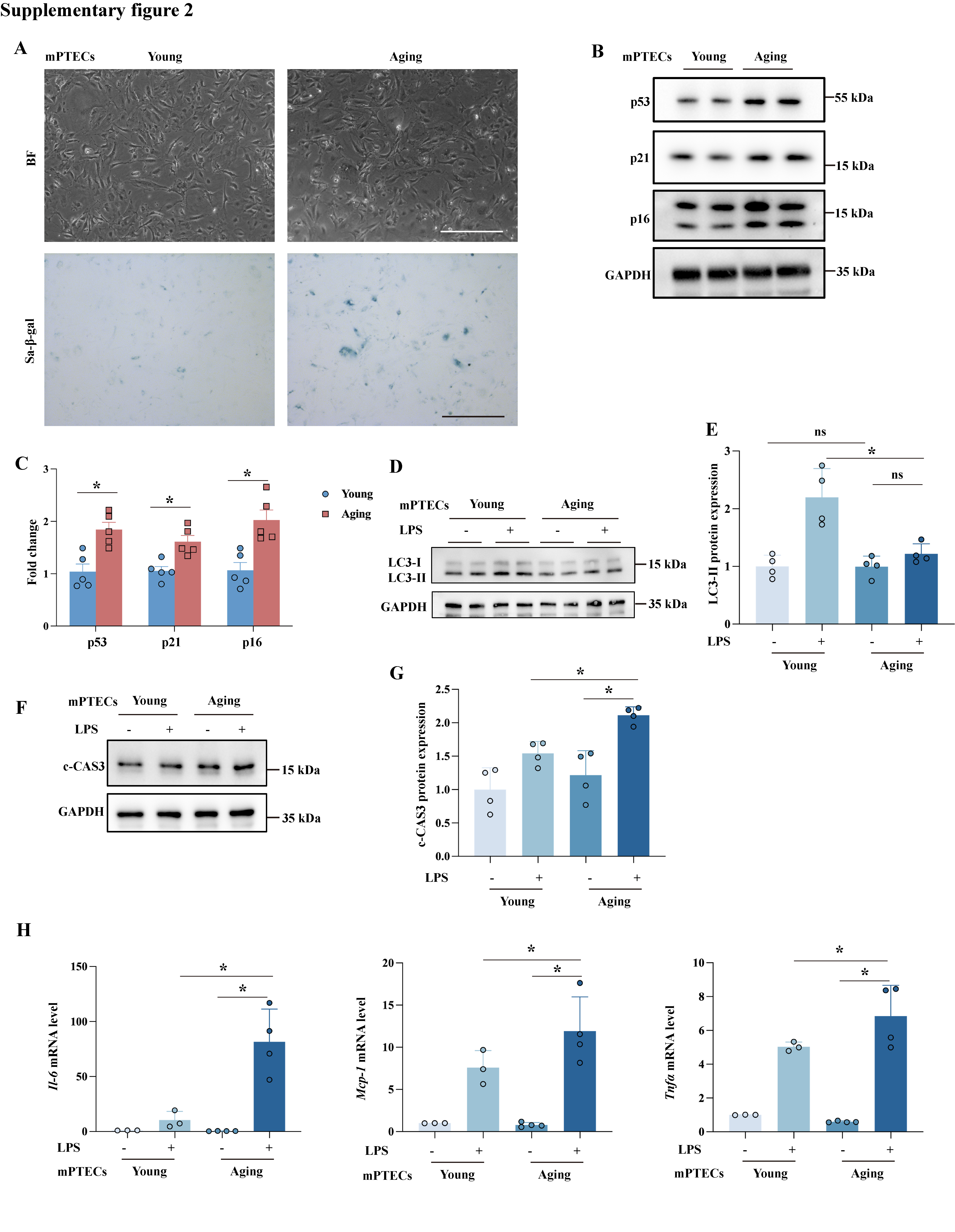
*P<0.05.

**Supplementary Figure 2. Primary renal tubular cells of old mice cultured in vitro are more sensitive to LPS and have dysregulated autophagy than young mice.**

(A-H) mPTCEs were extracted from aging (18 months) and young (2 months) male C57BL/6 mice cultured in complete medium of DMEM/F12 containing 10% FBS. Cells were treated with 10 μg/ml LPS and cell samples were collected after 24 hours. (A) SA-β-gal staining of mPTCEs from aging and young mouse, bar scale = 50 μm. (B) Representative immunoblots of P53, P21, P16 and GAPDH expression of mPTCEs from aging and young mouse, and (C) corresponding densitometric quantification. (D) Representative immunoblots of p53, p21, p16 and GAPDH expression, and (E) corresponding densitometric quantification. (F) Representative immunoblots of c-CAS3 and GAPDH expression, and (G) corresponding densitometric quantification. (H) Relative mRNA expression levels of the genes encoding *Il-6, Mcp-1* and *Tnfα*, assessed by RT-qPCR. All quantitative data are expressed as mean ± SEM. *P<0.05.

**Supplementary Figure**
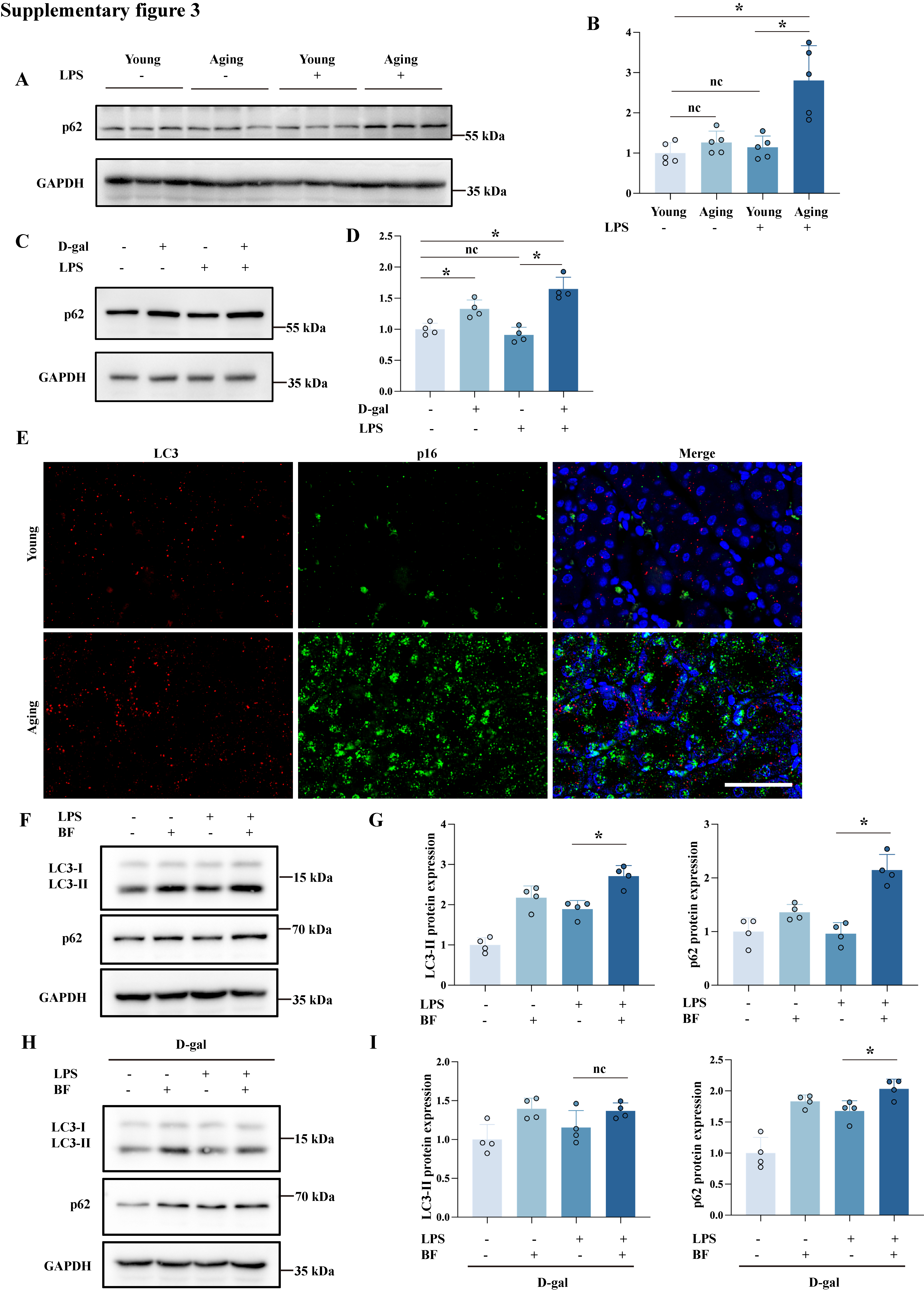
**3.** **Autophagy dysfunction in senescent renal tubular epithelial cells.** (A–B) Aging (18 months) and young (2 months) male C57BL/6 mice were injected with 10 mg/kg LPS (LPS group) or saline vehicle as control (Con group) to collect samples for analysis 12 hours later. (A)Representative immunoblots of p62 and GAPDH expression in kidney tissues, and (B) corresponding densitometric quantification of p62. (C-D) BUMPT cells were treated with D-gal (300 mM) for 72 hours and then with LPS (10 μg/ml) for 24 hours. (C)Representative immunoblots of p62 and GAPDH expression in BUMPT cells, and (D) corresponding densitometric quantification of p62. (E) Representative images of LC3B immunofluorescence (Red), p16 immunofluorescence (Green) and the nucleus (Hoechst, blue) in kidney tubules; bar scale = 50 μm. (F-I) Normal BUMPT cells and D-gal-induced senescent BUMPT cells were exposed to LPS for 24 hours, followed by treatment with Bafilomycin A1 for an additional 4 hours before sample collection. (F) Representative immunoblots of LC3-I/II, p62 and GAPDH expression, and (G) corresponding densitometric quantification of LC3-II, p62. (H) Representative immunoblots of LC3-I/II, p62 and GAPDH expression in kidney tissues, and (I) corresponding densitometric quantification of LC3-II, p62. All quantitative data are expressed as mean ± SEM. *P<0.05.


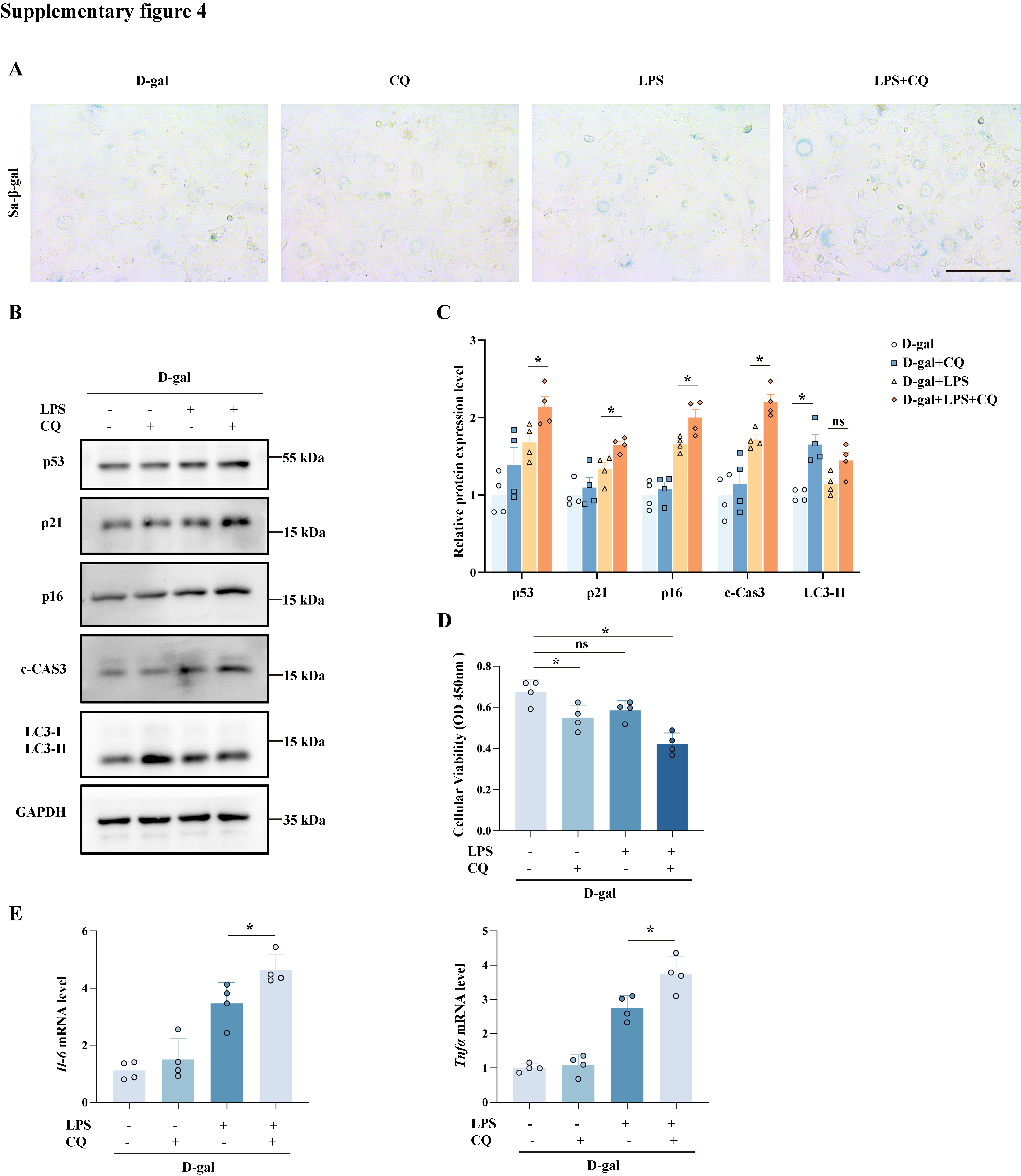


**Supplementary Figure 4. Autophagy inhibition aggravates LPS-induced injury and senescence burden in senescent BUMPT cells.** (A-E) Treatment of LPS-treated senescent BUMPT cells with chloroquine (20 μm). (A) SA-β-gal staining of mPTCEs from aging and young mouse, bar scale = 50 μm. (B) Representative immunoblots of p53, p21, p16, c-CAS3, LC3-I/II, and GAPDH expression, and (C) corresponding densitometric quantification of p53, p21, p16, c-CAS3 and LC3-II. (D) Cell viability assessed by CCK-8 assay. (E) Relative mRNA expression levels of the genes encoding *Il-6,* and *Tnfα*, assessed by qRT-qPCR. All quantitative data are expressed as mean ± SEM. *P<0.05.


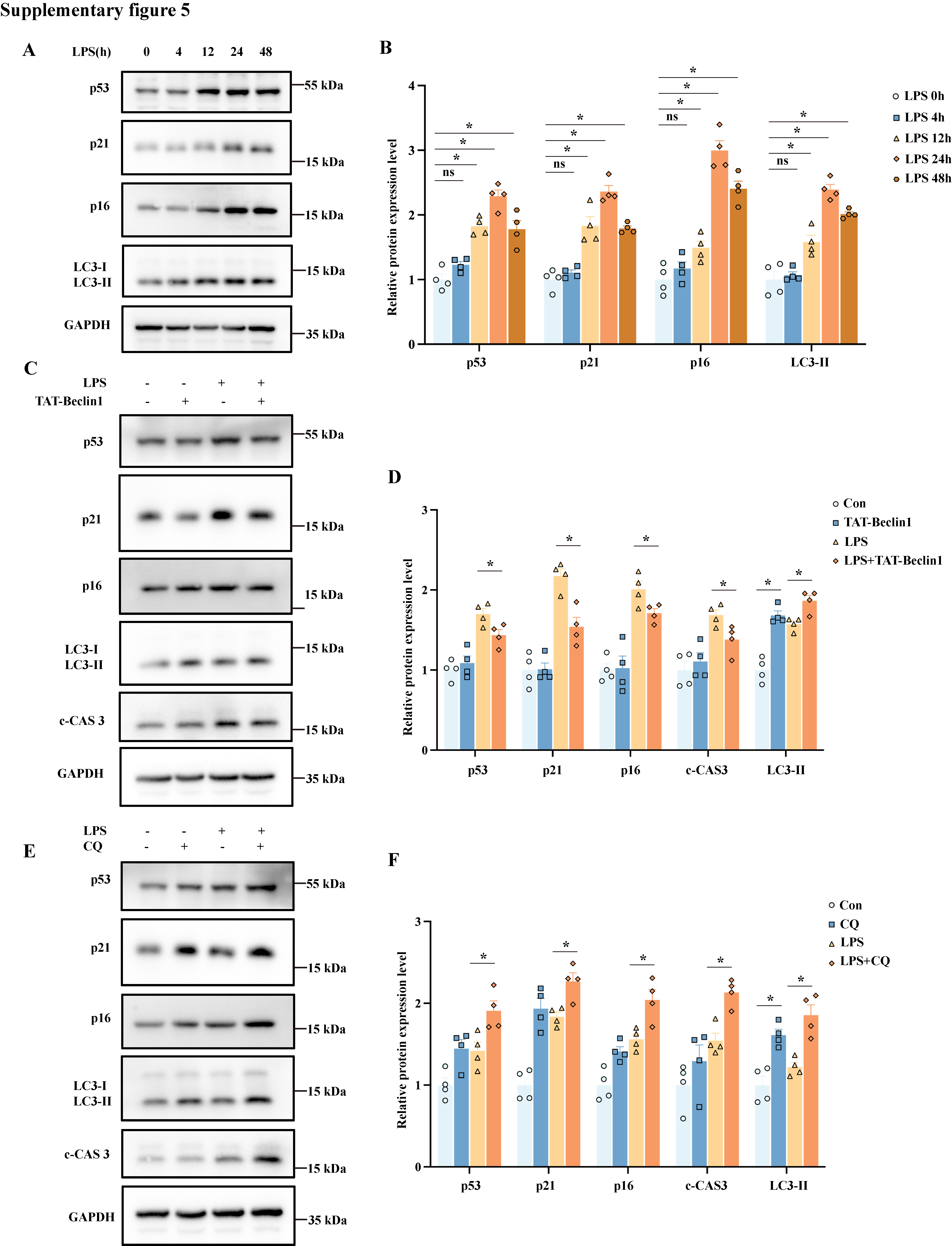


**Supplementary Figure 5. Autophagy modulation influences the senescence phenotype of LPS-treated BUMPT cells.** (A-B) BUMPT cells cultured in vitro was treated with LPS for 0, 4, 12, 24 and 48 hours, respectively. (A) Representative immunoblots of p53, p21, p16, LC3-I/II, and GAPDH expression, and (B) corresponding densitometric quantification of p53, p21, p16, LC3-II. (C-F) Treatment of LPS-treated BUMPT cells with Tat-Beclin 1 peptide (30 μm) and chloroquine (20 μm), respectively. (C) Representative immunoblots of p53, p21, p16, c-Cas3, LC3-I/II, and GAPDH expression, and (D) corresponding densitometric quantification of p53, p21, p16, c-Cas3 and LC3-II. (E) Representative immunoblots of p53, p21, p16, c-Cas3, LC3-I/II, and GAPDH expression, and (F) corresponding densitometric quantification of p53, p21, p16, c-Cas3 and LC3-II. All quantitative data are expressed as mean ± SEM. *P<0.05.


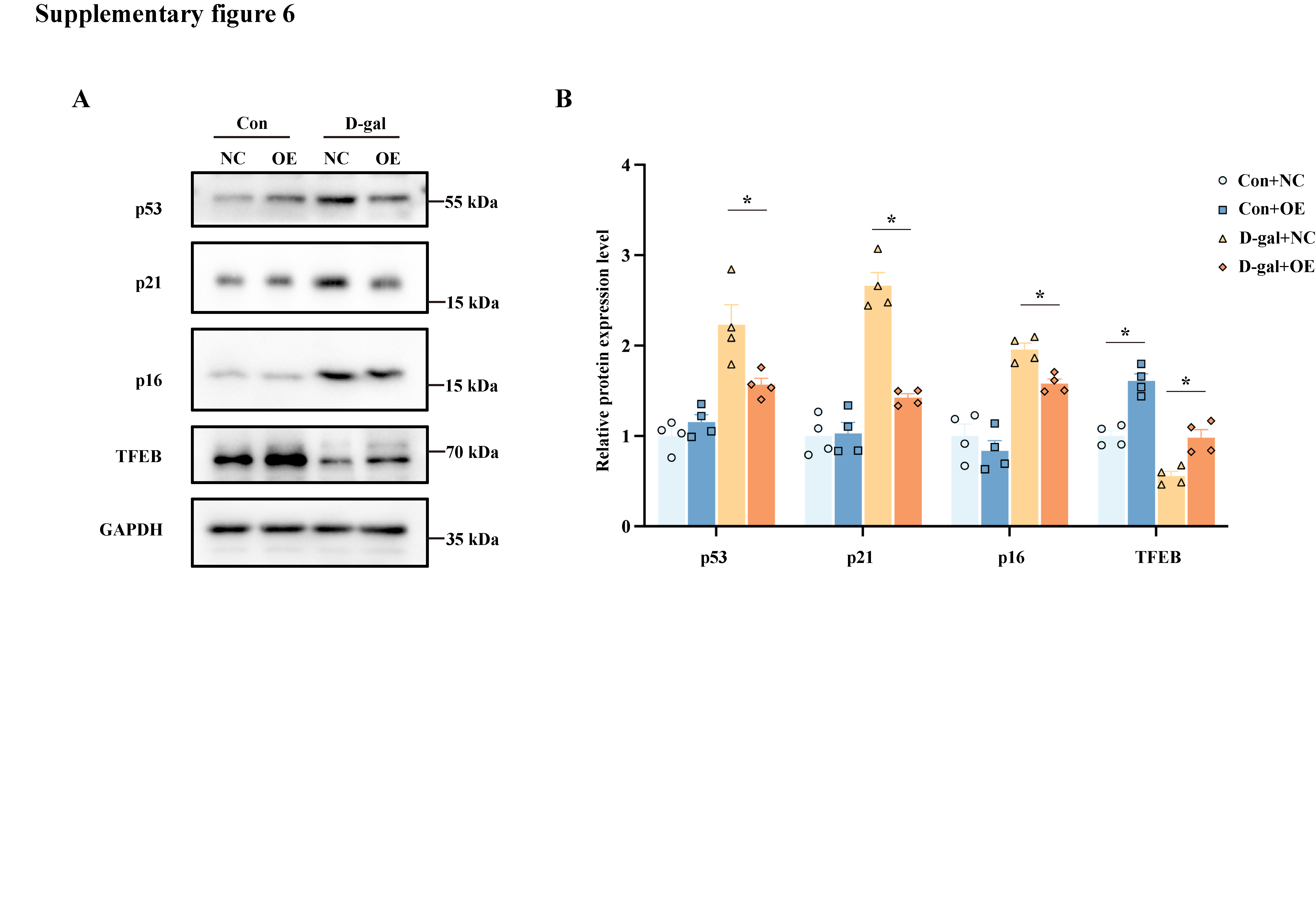


**Supplementary Figure 6. Overexpression of TFEB alleviates D-gal-induced senescence in BUMPT cells.** (A-B) BUMPT cells were transfected with a TFEB overexpression plasmid (OE) or an empty vector control (NC). After 7 hours, the medium was replaced, and D-gal (300 mM) was added for 72 hours to induce cellular senescence. (A) Representative immunoblots of p53, p21, p16, TFEB and GAPDH expression, and (B) corresponding densitometric quantification of p53, p21, p16, c-Cas3 and TFEB. All quantitative data are expressed as mean ± SEM. *P<0.05.


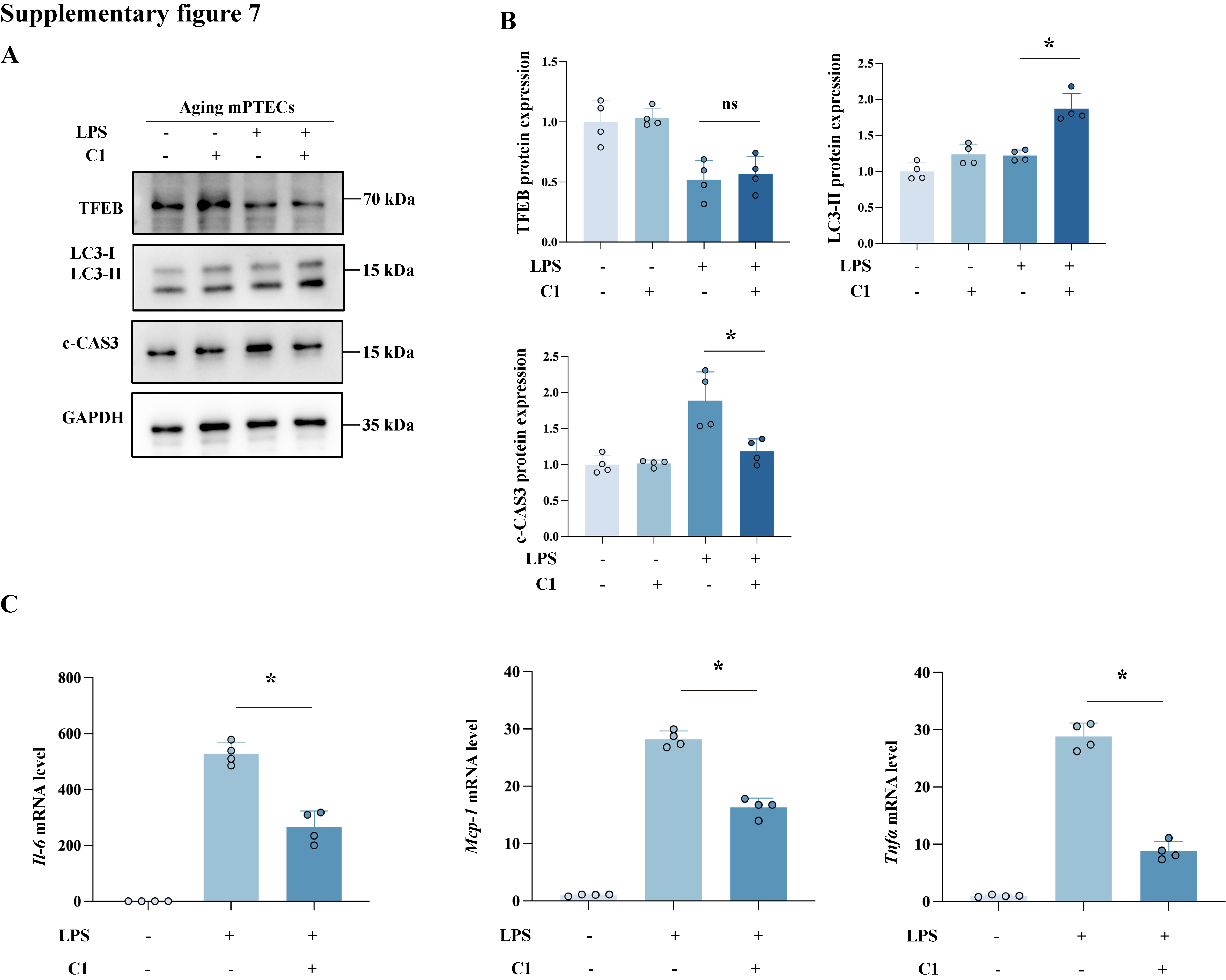


**Supplementary Figure 7.** **Promoting the nuclear translocation of TFEB can protect LPS-induced apoptosis and inflammation by activating autophagy in mPTECs in vivo.**

(A-C) mPTECs were extracted from aging (18 months) male C57BL/6 mice cultured in complete medium of DMEM/F12 containing 10% FBS. Cells were collected after 24 hours of LPS (10ug/ml) treatment. (A) Representative immunoblots of TFEB, LC3-I/II, c-CAS3 and GAPDH expression in BUMPT cells, and (B) corresponding densitometric quantification. (C) Relative mRNA expression levels of the genes encoding *Il-6, Mcp1* and *Tnfα*, assessed by qRT-qPCR. All quantitative data are expressed as mean ± SEM. *P<0.05.
